# Global reference mapping of human transcription factor footprints

**DOI:** 10.1038/s41586-020-2528-x

**Published:** 2020-07-29

**Authors:** Jeff Vierstra, John Lazar, Richard Sandstrom, Jessica Halow, Kristen Lee, Daniel Bates, Morgan Diegel, Douglas Dunn, Fidencio Neri, Eric Haugen, Eric Rynes, Alex Reynolds, Jemma Nelson, Audra Johnson, Mark Frerker, Michael Buckley, Rajinder Kaul, Wouter Meuleman, John A. Stamatoyannopoulos

**Affiliations:** 1 Altius Institute for Biomedical Sciences, Seattle, WA USA; 2 Department of Genome Sciences, University of Washington, Seattle, WA USA; 3 Division of Oncology, Department of Medicine, University of Washington, Seattle, WA USA

**Keywords:** Epigenomics, Functional genomics, Gene regulation, Epigenomics

## Abstract

Combinatorial binding of transcription factors to regulatory DNA underpins gene regulation in all organisms. Genetic variation in regulatory regions has been connected with diseases and diverse phenotypic traits^1^, but it remains challenging to distinguish variants that affect regulatory function^2^. Genomic DNase I footprinting enables the quantitative, nucleotide-resolution delineation of sites of transcription factor occupancy within native chromatin^3–6^. However, only a small fraction of such sites have been precisely resolved on the human genome sequence^6^. Here, to enable comprehensive mapping of transcription factor footprints, we produced high-density DNase I cleavage maps from 243 human cell and tissue types and states and integrated these data to delineate about 4.5 million compact genomic elements that encode transcription factor occupancy at nucleotide resolution. We map the fine-scale structure within about 1.6 million DNase I-hypersensitive sites and show that the overwhelming majority are populated by well-spaced sites of single transcription factor–DNA interaction. Cell-context-dependent *cis*-regulation is chiefly executed by wholesale modulation of accessibility at regulatory DNA rather than by differential transcription factor occupancy within accessible elements. We also show that the enrichment of genetic variants associated with diseases or phenotypic traits in regulatory regions^1,7^ is almost entirely attributable to variants within footprints, and that functional variants that affect transcription factor occupancy are nearly evenly partitioned between loss- and gain-of-function alleles. Unexpectedly, we find increased density of human genetic variation within transcription factor footprints, revealing an unappreciated driver of *cis*-regulatory evolution. Our results provide a framework for both global and nucleotide-precision analyses of gene regulatory mechanisms and functional genetic variation.

## Abstract (web summary)

A high-density DNase I cleavage map from 243 human cell and tissue types provides a genome-wide, nucleotide-resolution map of human transcription factor footprints.

## Main

Genome-encoded recognition sites for sequence-specific DNA binding proteins are the atomic units of eukaryotic gene regulation. Currently we lack a comprehensive, nucleotide-resolution annotation of such elements and their selective occupancy in different cell types and states. Such a reference is essential both for analysis of cell-selective regulation and for systematic integration of regulation with genetic variation associated with diseases and phenotypic traits.

In vivo binding of regulatory factors shields bound DNA elements from nuclease attack, giving rise to protected single-nucleotide-resolution DNA ‘footprints’. The advent of DNA footprinting using the non-specific nuclease DNase I^8^ marked a turning point in analyses of gene regulation, and facilitated the identification of the first mammalian sequence-specific DNA binding proteins^9^. Genomic DNase I footprinting^3–6^ enables the genome-wide delineation of DNA footprints (approximately 7–35 base pairs (bp)) over any genomic region in which DNase I cleavage is sufficiently dense—chiefly DNase I hypersensitive sites (DHSs). DNase I footprints pinpoint regulatory factor occupancy on DNA and can be used to discriminate sites of direct versus indirect occupancy when integrated with chromatin immunoprecipitation and sequencing (ChIP–seq) experiments^4^. Cognate transcription factors (TFs) can be assigned to footprints on the basis of matching consensus sequences, enabling the TF-focused analysis of gene regulation and regulatory networks^10^ and of the evolution of regulatory factor binding patterns^11^. DNase I is roughly the size of a typical TF and recognizes the minor groove of DNA, where it hydrolyses single-stranded cleavages. These, in turn, reflect both the topology and the kinetics of coincidently bound proteins. Previous efforts to analyse these featues^4^ were complicated by the slight sequence-driven cleavage preferences of DNase I, which have since been exhaustively determined^12^, setting the stage for fully resolved tracing of DNA–protein interactions within regulatory DNA.

Here we combine sampling of more than 67 billion uniquely mapping DNase I cleavages from over 240 human cell types and states to index human genomic footprints with unprecedented accuracy and resolution, and thereby to identify the sequence elements that encode TF recognition sites within the human genome. We leverage this index to (i) systematically assign footprints to TF archetypes; (ii) define patterns of cell-selective occupancy; and (iii) analyse the distribution and effect of human genetic variation on regulatory factor occupancy and the genetics of common diseases and traits.

## Global mapping of TF footprints

To create comprehensive maps of TF occupancy, we deeply sequenced high-quality, high-complexity DNase I libraries from 243 biosamples derived from diverse primary cells and tissues (*n* = 151), primary cells in culture (*n* = 22), immortalized cell lines (*n* = 10) and cancer cell lines and primary samples (*n* = 60) (Supplementary Table 1). Collectively, we uniquely mapped 67.6 billion DNase I cleavage events (mean, 278.2 million uniquely mapped cleavages per biosample), which represents a great increase over earlier studies^4^. On average, 49.7% of DNase cleavages from each biosample mapped to DHSs, which covered 1–3% of the genome.

**Extended Data Fig. 1:**
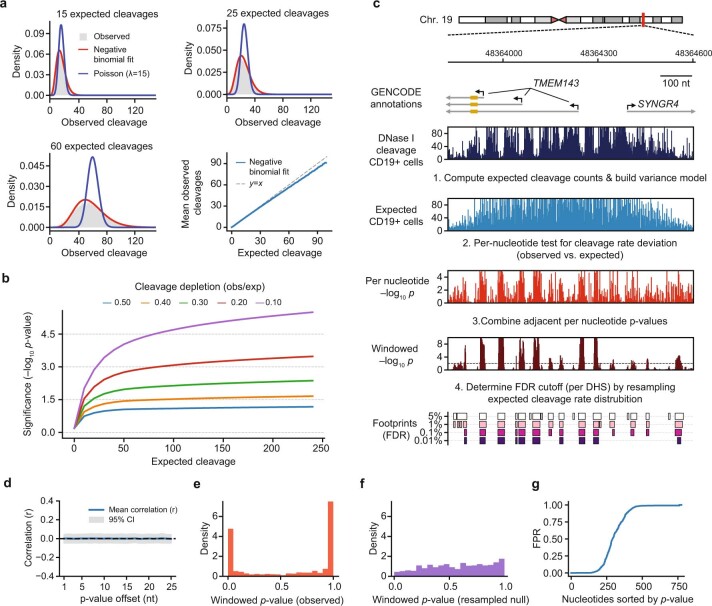
Statistical modelling of DNase I cleavage variation and footprint detection within a single dataset. **a**, A negative binomial model was fit from the distribution of observed cleavage counts for each predicted cleavage rate. Shown are histograms of observed cleavage counts in CD19^+^ B cells at all genomic sites with 5, 25, or 60 expected cleavages. Red, the negative binomial distribution fit to the observed data using maximum likelihood estimation (Supplementary Methods). Blue, Poisson distribution with *λ* set to the corresponding expected cleavage rate. Lower right panel, means of fitted negative binomial distributions vs means of observed cleavage rates. Dashed grey line indicates *y* = *x* for reference. **b**, Estimated power of empirical cleavage dispersion model. Computed *P* values for different cleavage rate effect sizes with respect to expected cleavage rates in CD19^+^ B cells. Coloured lines represent the modelled effect size (depletion of cleavages) relative to the expected rate corresponding to a hexamer sequence model. **c**, Example of footprint detection within promoters for *TMEM143* and *SYNGR4* in CD19^+^ B cells. Expected cleavages were generated by reassigning observed cleavages according to a hexamer cleavage model (Supplementary Methods). The significance of difference between the observed and expected cleavages was evaluated per nucleotide using the negative binomial dispersion model. Individual *P* values are combined in 7-bp windows using Stouffer’s *Z*-score method. Per-nucleotide false discovery rates were computed by sampling from the expected null distributions. **d**, Autocorrelation of *P* values sampled from the expected negative binomial distribution. **e**, **f**, Histogram of windowed *P* values for observed (**e**) and sampled (**f**) data. **g**, Observed and sampled *P* values compared to empirically determine and calibrate false-positive rates (FPR).

To identify DNase I footprints genome-wide, we developed a computational approach that incorporates both chromatin architecture and exhaustively enumerated empirical DNase I sequence preferences to determine expected per-nucleotide cleavage rates across the genome, and to derive, for each biosample, a statistical model for testing whether its observed cleavage rates at individual nucleotides deviated significantly from expectation (Extended Data Fig. 1a–g, Supplementary Methods). We note that the derivation of cleavage variability models for each biosample individually accounts for additional sources of technical variability beyond DNase I cleavage preference.

**Extended Data Fig. 2:**
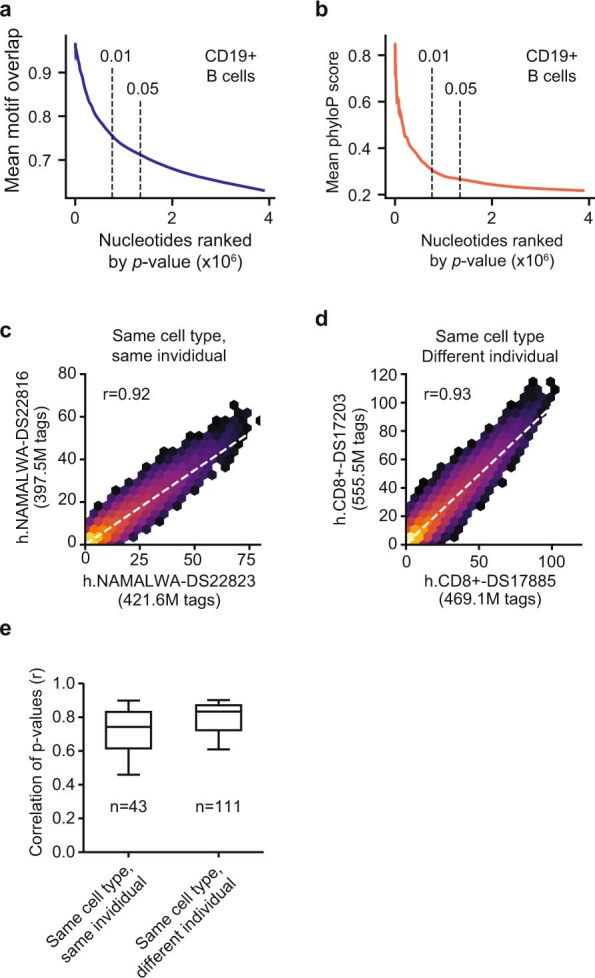
Genomic footprints are reproducible, overlap evolutionarily constrained nucleotides, and are enriched for TF recognition sequences. **a**, Motif density is associated with footprint strength. Plotted is the overlap of motif recognition sequence matches (*P* < 0.0001) with nucleotides ranked by footprint *P* value from CD19^+^ B cells. **b**, As in **a**, but for per-nucleotide evolutionary conservation (phyloP). **c**, Scatter plot of per-nucleotide footprint *P* values for replicate experiments from the same cell line (NAMALWA Burkitt’s lymphoma cells). All individual nucleotides within FDR 1% footprints in either replicate were considered for correlation analysis. **d**, As in **c**, but for replicates of the same primary cell (CD8^+^ T cells) between two distinct individuals. **e**, Pearson’s correlation between replicates pairs grouped by whether they were derived from the same cell and individual (*n* = 43) or were the same primary cell or tissue from different individuals (*n* = 111). Boxes indicate median and inner quartile-range (IQR). Whiskers, 5th and 95th percentile.

Using this model, we performed de novo footprint discovery independently on each of 243 biosamples, detecting on average 657,029 high-confidence footprints per biosample (range 220,580–1,664,065, empirical false discovery rate <1% (Supplementary Methods)), and collectively 159.6 million footprint events across all biosamples. Nucleotide protection tracked closely with both the presence of known TF recognition sequences and the level of per-nucleotide evolutionary conservation (Extended Data Fig. 2a, b). At the level of individual nucleotides, de novo footprints genome-wide were highly concordant between biological replicates of the same cultured cell type or between the same primary cell and tissue types sampled from different individuals (median Pearson’s *r* = 0.83 and 0.74, respectively) (Extended Data Fig. 2c–e). Within each biosample, footprints encompassed an average of around 7.6 Mb (0.2%) of the genome, with a mean of 4.3 footprints per DHS with sufficient read depth for robust detection (normalized cleavage density within DHS of at least 1).

## Unified index of human genomic footprints

Comparative footprinting across cell types has the potential to illuminate both the structure and function of regulatory DNA, but a systematic approach for joint analysis of genomic footprinting data has been lacking. Given the scale and diversity of the cell types and tissues surveyed, we sought to develop a framework that could integrate hundreds of available footprinting datasets to increase the precision and resolution of footprint detection and, furthermore, to provide a scaffold for a common reference index of TF-contacted DNA genome-wide.

**Fig. 1:**
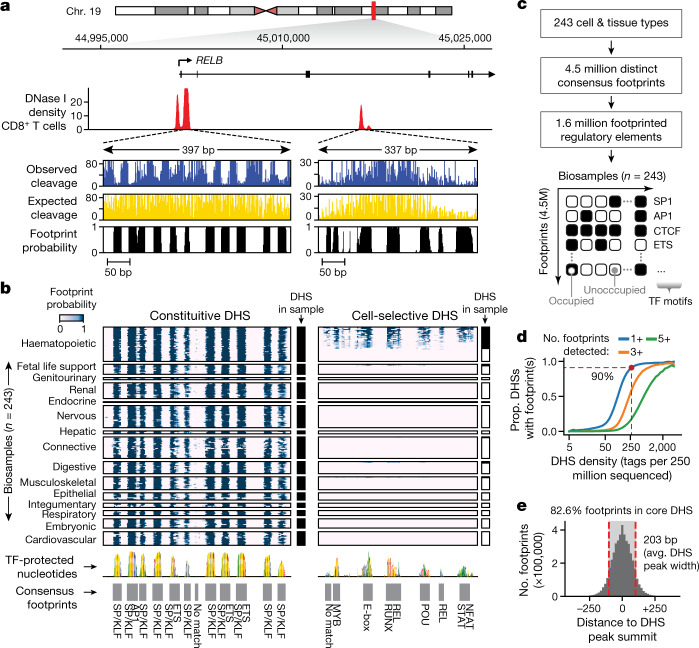
A nucleotide-resolution atlas of TF occupancy on the human genome. **a**, DNase I cleavage patterns (*RELB* locus in CD8^+^ T cells). Top, windowed DNase I cleavage density. Below, per-nucleotide cleavage and footprint posterior probabilities within two DHSs. **b**, Heat map of footprint posterior probabilities integrating 243 biosamples. Rows are individual biosamples grouped by tissue or organ systems; columns are individual nucleotides. Black fills to right of heat maps indicate overlapping DHSs in biosample. Below, DHS sequence scaled by footprint prevalence. Grey boxes, consensus footprints in one or more cell or tissue types (footprint posterior >0.99). **c**, Consensus map of TF occupancy derived from 243 biosamples covering 1.6 million DHSs providing expansive nucleotide-resolution annotation of regulatory DNA. **d**, Proportion of DHSs with footprints at given sequencing depth. Dashed red lines and dot show read depth (tags per 250 million uniquely mapped reads) at which footprint is detected in 90% of DHSs. **e**, Histogram of footprint location relative to DHS peak summit. Dashed red lines represent average size of a DHS peak (203 bp).

To accomplish this, we implemented an empirical Bayes framework that estimates the posterior probability that a given nucleotide is footprinted by incorporating a prior on the presence of a footprint (determined by footprints independently identified within individual datasets) and a likelihood model of cleavage rates for both occupied and unoccupied sites (Fig. 1a, Supplementary Methods). Figure 1b depicts per-nucleotide footprint posterior probabilities computed for two DHSs within a representative locus (*RELB*) across all 243 biosamples. A notable feature of these data is the positional stability and discrete appearance of footprints seen within each DHS across tens to hundreds of biosamples. Plotting individual nucleotides scaled by their footprint prevalence across all samples precisely resolves the core recognition sequences of diverse TFs (Fig. 1b, bottom).

**Extended Data Fig. 3:**
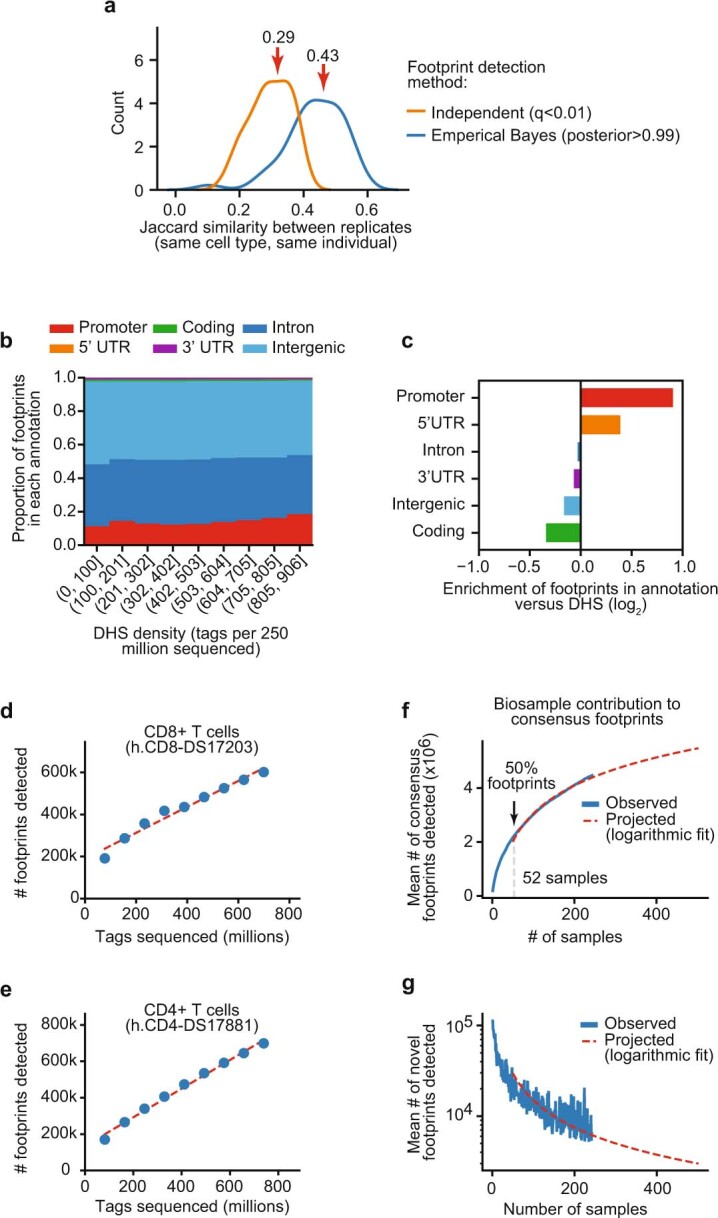
Characteristics of consensus derived genomic footprints. **a**, Histogram of footprint discovery concordance (Jaccard similarity) between replicates (same cell type, same individual) using footprints called either independently (FDR 1%) (light orange) or with the empirical Bayes approach (posterior probability >0.99). **b**, Genomic distribution of consensus footprints stratified by DNase I signal of the encompassing DHS. **c**, Enrichment of footprint overlap in gene annotations vs. the distribution of DHSs. **d**, Effect of sequencing depth on footprint detection in CD8^+^ T cells. Sequencing tags were randomly subsampled (90% down to 10%) from the complete library. Footprints were called independently on each subsampled set of tags. Plotted is the number of FDR 1% footprints detected vs subsampled sequencing depth. Red dashed line, linear model fit. **e**, Same as **d** for CD4^+^ T cells. **f**, **g**, Contribution of individual samples to the consensus footprint index. Datasets were ordered randomly, and the collective number of footprints was computed after the footprints present within each additional dataset was considered. **f**, Mean total number of consensus footprints detected vs. number of datasets included after 100 iterations of random dataset orderings. Red dashed line shows a logarithmic curve fit (excluding first 50 samples). Grey dashed line indicates number of datasets that recapitulate 50% of consensus footprints. **g**, Mean number of novel footprints detected after the sequential addition of each sample.

To establish a reference set of TF-occupied DNA elements genome-wide, we applied the Bayesian approach to all DHSs detected within one or more of the 243 biosamples, and applied the same consensus approach used to establish a consensus DHS index^13^ to collate overlapping footprinted regions across individual biosamples into distinct high-resolution consensus footprints (Supplementary Methods). Collectively, we delineated approximately 4.46 million consensus footprints within about 1.6 million (46%) of the 3.39 million DHSs indexed within these biosamples^13^ (Fig. 1c). More than 90% of the DHSs with moderate sequencing coverage (over 250 tags per 250 million sequenced) contained at least one footprint (and typically many more; Fig. 1d). As expected, consensus (that is, empirical Bayes) footprints were markedly more reproducible than footprints detected using individual datasets (average Jaccard similarity between replicate biosamples 0.43 versus 0.29, respectively) (Extended Data Fig. 3a).

Consensus footprints were on average 16 bp wide (middle 95%: 7–44 bp; 90%: 7–36 bp; 50%: 9–21 bp) and were distributed across all classes of DHS, albeit with enrichment in promoter-proximal elements owing to their generally elevated cleavage density (Extended Data Fig. 3b, c). Most consensus footprints (82.6%) localized directly within the core of a DHS peak (average width 203 bp), with virtually all of the remainder localized within 250 bp of a DHS peak summit (Fig. 1e). Collectively, consensus footprints annotated 2.1% (72 Mb) of the human genome reference sequence, compared with about 1.5% for protein coding elements.

Given the strong dependency of footprint detection on sequencing depth (Extended Data Fig. 1b) and sample diversity, we sought to estimate how comprehensively this index covered the possible detectable footprint space and to what degree additional sequencing and/or biosamples would augment footprint discovery. De novo footprint detection after iteratively subsampling the most deeply sequenced DNase I libraries (more than 750 million sequenced tags) showed that footprints detected increased linearly with sequencing depth (Extended Data Fig. 3d, e), indicating that these DNase I libraries have yet to be sampled to saturation. By contrast, the addition of new biosamples and/or replicates produced a sublinear increase in the number of footprints detected (Extended Data Fig. 3f, g). Because the consensus approach favours footprints with support from many biosamples, the consensus footprint space reported here is likely to represent a substantial proportion of TF binding sites that are shared across many cell and tissue types.

## Assigning TFs to footprints

**Extended Data Fig. 4:**
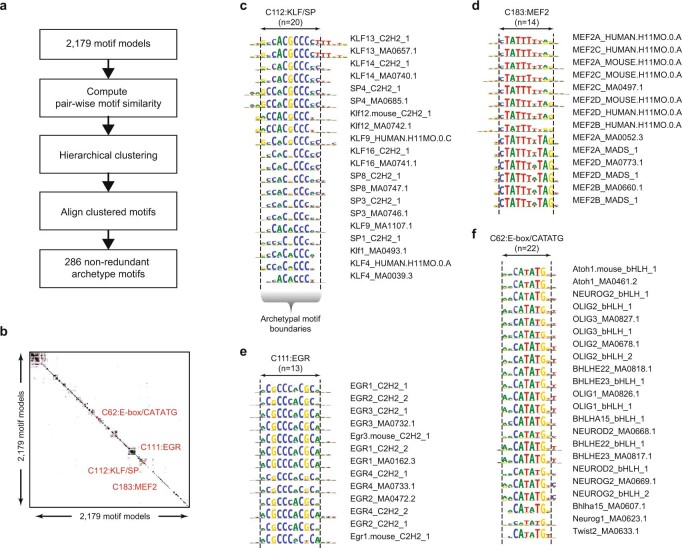
Clustering motifs by similarity. **a**, Outline of motif clustering approach. Motif models (*n* = 2,174) from ref. ^11^, JASPAR^12^ (2018), and HOCOMOCO^13^ were clustered by motif similarity. **b**, Hierarchically clustered heat map of the pairwise similarity scores between motifs. The cluster dendrogram was cut at height 0.7 to create non-redundant archetypal clusters of motifs. **c**–**f**, Exemplar clusters of similar TF recognition sequences corresponding to KLF/SP (C2H2 family), EGR (C2H2 family), MEF2 (MADS) and E-box/CATATG (bHLH).

Recognition sequences now exist for all major families and subfamilies of TFs, and for a large number of individual TF isoforms^14^. We thus sought to create a reference mapping between annotated TFs and consensus footprints by (i) compiling and clustering all publicly available motif models^15–17^; (ii) creating non-redundant TF archetypes by placing closely related TF family members on a common sequence axis (Extended Data Fig. 4, Supplementary Table 2, Supplementary Methods); (iii) aligning TF archetypes to the human reference sequence at high stringency (*P* < 10^−4^); and (iv) enumerating all potential TF archetypes that are compatible with each consensus footprint on the basis of overlap and match stringency. In total, 80.7% of the approximately 4.46 million consensus footprints could be assigned to at least one TF with at least 90% sequence overlap, of which 860,780 (19.3%) could be unambiguously assigned to a single factor, and 2,038,220 (45.7%) to a single TF with two lower-ranked alternatives.

**Extended Data Fig. 5:**
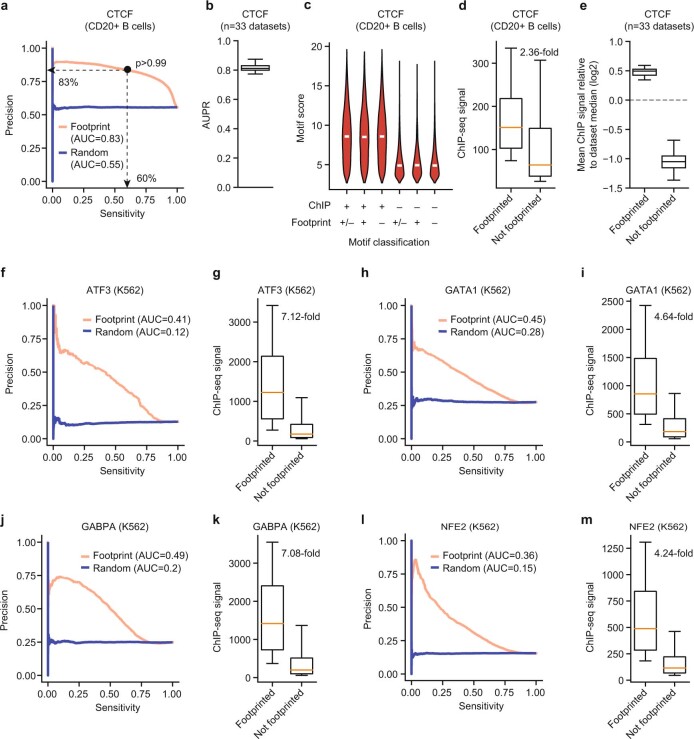
Classification of ChIP–seq data by genomic footprinting. **a**, Precision-recall (PR) curve for predictions of CTCF motif occupancy (that is, overlap CTCF ChIP–seq peak) based on footprint posterior probabilities in CD20^+^ B cells. Black dot indicates precision and recall at posterior footprint probability threshold of >0.99. Blue, PR curve computed after shuffling ChIP–seq peak labels. **b**, Area under precision-recall curve (AUPR) computed for 21 ENCODE cell types and/or replicates (*n* = 33 total datasets). **c**, Distribution of MOODS scores stratified by motif overlap with a ChIP–seq peak and/or a genomic footprint in CD20^+^ B cells. **d**, ChIP–seq signal intensity at peaks overlapping a footptrinted CTCF motif vs. non-footprinted motifs in CD20^+^ B cells. **e**, Relative ChIP-seq signal at footprinted and non-footprinted CTCF peaks containing a motif across 21 cell types (*n* = 32 total datasets). **f**–**m**, PR curves and relative ChIP–seq intensities for ATF3 (**f**, **g**), GATA1 (**h**, **i**), GABPA (**j**, **k**) and NFE2 (**l**, **m**) in K562 cells. For all TFs analysed, only motifs overlapping DHSs were considered. DHS, ChIP-seq and motif models are described in Supplementary Table 3. Boxes indicate median and IQR. Whiskers, 5th and 95th percentiles.

To gauge the sensitivity and accuracy of the motif-to-consensus footprint mappings, we evaluated the posterior footprint probability as metric to classify motif occupancy by using the genomic master regulator CCCTC-binding factor (CTCF). CTCF combines a well-documented, unambiguous motif with the availability of ENCODE ChIP–seq data^18^ for a broad range of cell and tissue types that match those represented in the consensus footprint index (Supplementary Table 3). Comparing the occupancy of all CTCF motifs within all DHSs (Supplementary Methods) with CTCF ChIP–seq data showed strong classification performance, with a mean area under precision-recall curve of 0.80 (Extended Data Fig. 5a, b). At the posterior footprint probability threshold used to generate consensus footprints (*P* > 0.99), we correctly identified an average of 19,904 CTCF-bound recognition elements per cell type, corresponding to a mean precision of 82.5% and sensitivity of 60% (Supplementary Table 3), despite posterior footprint probability not encoding any information about the quality of motif matches. Lower CTCF motif match scores were strongly associated with false-positive footprint or motif classifications, so the incorporation of motif match strength in addition to footprint probability is expected to increase classification precision (Extended Data Fig. 5c). Overall, footprinted motifs showed an approximately 2.5-fold increase in CTCF ChIP–signal when compared to non-footprinted motifs (Extended Data Fig. 5d, e). Examination of other TFs yielded similar results, albeit with variable classification accuracy that was probably driven by the ambiguity in footprint assignment for motifs recognized by many distinct TFs and the predominance of weak and/or indirect occupancy (Extended Data Fig. 5f–m).

## Primary architecture of regulatory regions

**Fig. 2:**
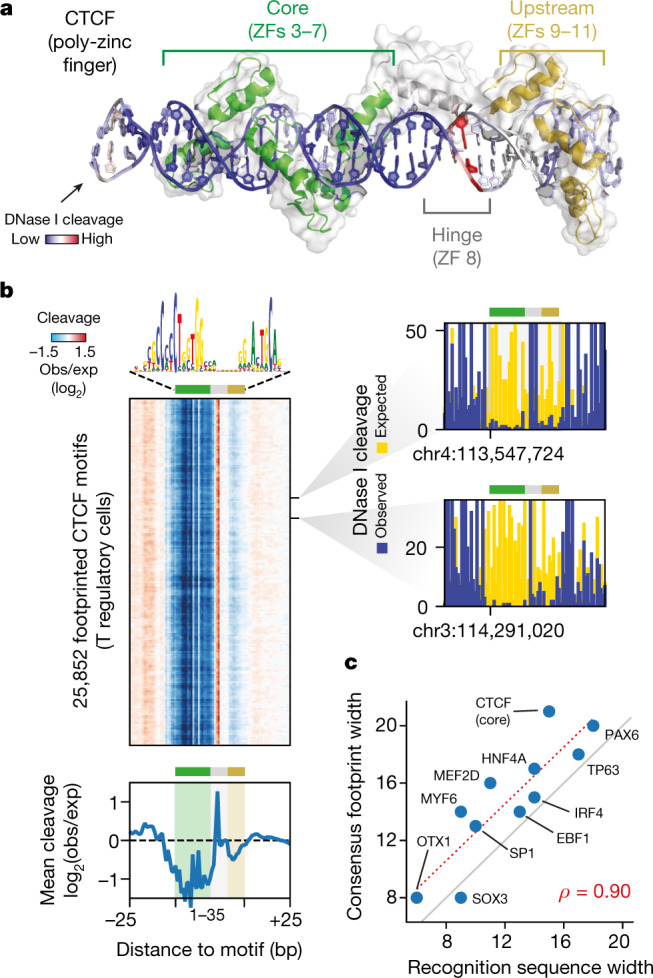
Footprints encapsulate topological structures of individual TF–DNA interactions. **a**, Structure of CTCF zinc fingers 3–11 bound to cognate DNA recognition sequence (Protein Data Bank (PDB) codes: 5YEF and 5YEL)^21^. DNA coloration shows mean observed versus expected cleavage at footprinted CTCF motifs (in T regulatory cells). **b**, Heat map of relative cleavage at each of 25,852 footprinted CTCF motifs (posterior probability >0.99). Below, aggregate (summed) nuclease cleavage relative to footprinted motifs. Right, nuclease cleavage (observed and expected) at three footprints randomly selected across genome. **c**, Footprint width is tightly correlated with the width of the TF recognition sequence (Spearman’s *ρ* = 0.9, *P* = 0.001).

**Extended Data Fig. 6:**
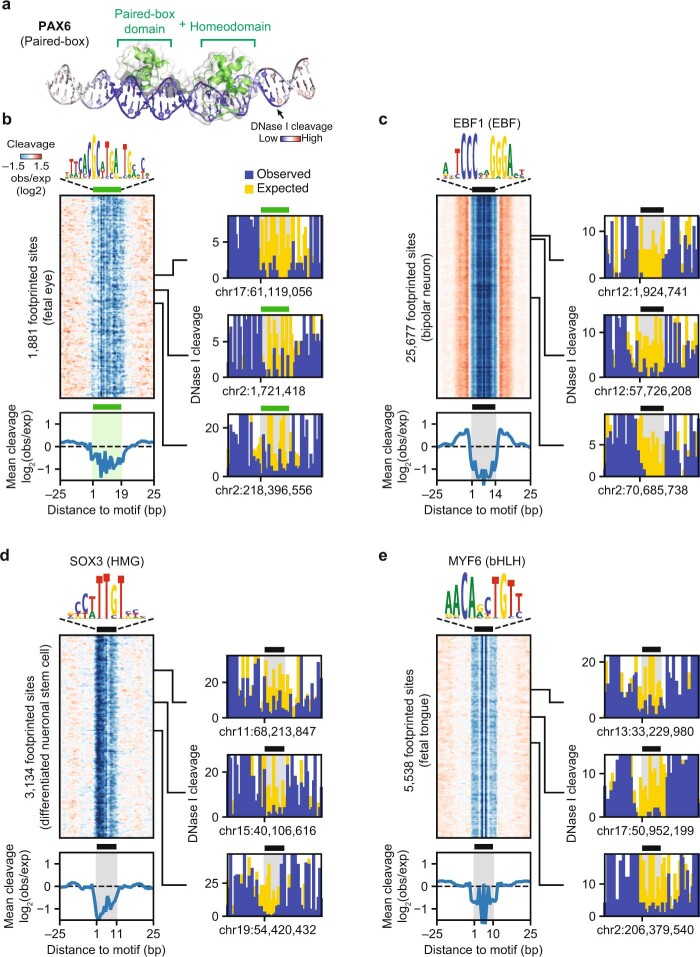
Aggregate DNase I cleavage profiles for diverse TFs. **a**, Physical structure of the paired-box TF PAX6 bound to its cognate recognition element (PDB: 6PAX)^24^. **b**–**e**, Per-nucleotide DNase I cleavage patterns surrounding instances of motifs within genomic footprints for PAX6 (fetal eye) (**b**); EBF1 (differentiated bipolar neuron) (**c**); SOX3 (differentiated neuronal cell) (**d**); and MYF6 (fetal tongue) (**e**). For each, top left shows a randomly ordered heat map of the per-nucleotide relative DNase I cleavage protection (observed/expected) for each footprinted motif instance (posterior footprint probability >0.99). Below, aggregate DNase I protection averaged over all footprinted sites. Right, DNase I cleavage at individual motif instances (blue, observed cleavage; yellow, expected cleavage).

Despite intensive efforts over several decades, the primary architecture of regulatory regions has remained unclear, with the singular exception of the interferon ‘enhanceosome’^19^. Elucidating the primary architecture of active regulatory DNA requires accurate tracing of the TF–DNA interface over an extended interval. Because TF engagement creates subtle alterations in DNA shape and protects underlying phosphate bonds from nuclease attack via steric hindrance^6^, we investigated to what extent fluctuations in corrected DNase I cleavage rates within individual consensus footprints accurately reflected the topology of the TF–DNA interface. Notably, previous efforts to resolve such features^4^ were obscured by subtle intrinsic cleavage preferences and lacked resolving power at individual TF footprints on the genome. Poly-zinc fingers are the most prevalent class of human TFs and have recognition interfaces that potentially cover tens of nucleotides^14^. The DNA recognition domain of CTCF comprises 11 zinc fingers, potentially encoding 33 bp of sequence (or DNA shape^20^) recognition. We identified 25,852 footprints that coincided precisely with CTCF motifs within regulatory T cells. Transposing the average corrected per-nucleotide cleavage propensity with an extended co-crystal structure of CTCF^21^ accurately traced all features of the protein–DNA interaction interface, including focal hypersensitivity within the hinge region between zinc fingers 7 and 9^5,22,23^ (Fig. 2a, Supplementary Methods). A similar result was obtained for widely divergent classes of DNA binding domain, such as the paired-box domain-containing TF PAX6^24^ (Extended Data Fig. 6a) and other TFs with extant co-crystal structures (not shown). Critically, these topological features were evident at the level of individual TF footprints on the genome (Fig. 2a, Extended Data Fig. 6). Overall, the average footprint width for diverse TFs tightly tracked the width of their respective recognition sequences (Spearman’s *ρ* = 0.90, *P* = 0.001) (Fig. 2b). As such, the extended profile of corrected per-nucleotide DNase I cleavage across entire regulatory regions should, in principle, provide a snapshot of the primary structure of active regulatory DNA.

## Distinguishing TF occupancy modes

TFs compete cooperatively with nucleosomes for access to regulatory DNA^25,26^. Many TFs have the potential to catalyse changes in nucleosome occupancy over a strongly matching recognition motif, a process referred to as ‘pioneering’^27^. However, it is unclear how steady-state chromatin accessibility is maintained by TFs in place of a canonical nucleosome, and whether this results primarily from local protein–protein interactions or the synergistic effects of independent TF–DNA binding^26^. We reasoned that the number, relative spacing, and morphology of TF binding events within individual regulatory elements could be used to gain insight into the mechanistic basis of TF cooperativity.

**Fig. 3:**
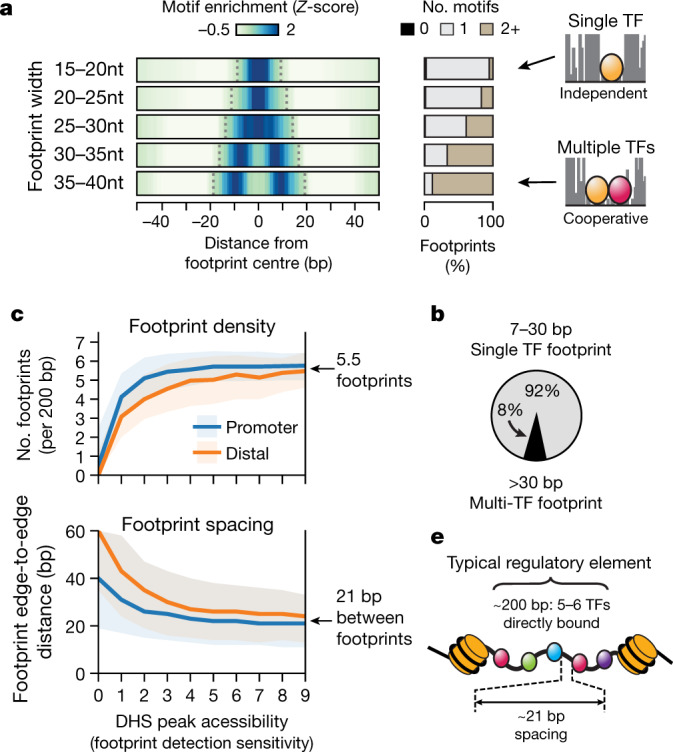
Modes of TF occupancy within regulatory DNA. **a**, Overlap and spatial enrichment of TF recognition sequences within footprints binned by width. Left, density heat map of motif occurrences around footprints binned by width. Right, proportion of footprints uniquely overlapped by 0, 1 or 2 or more recognition sequences. **b**, Percentage of footprints representing occupancy of single TF (≤30 bp) or multiple TFs (>30 bp). **c**, **d**, Footprint density and spacing (edge-to-edge) plotted against mean normalized cleavage density (tags per 150 bp per million reads) within promoter-proximal and distal DHSs. Solid lines and shaded regions indicate median and middle 50th percentile, respectively. **e**, A typical DHS contains about five or six directly bound TFs spaced roughly 20 bp apart.

As the width of genomic footprints tightly tracks the physical structure of individual TFs bound to DNA (Fig. 2a, b, Extended Data Fig. 6), and direct TF–TF interactions are dependent on close proximity, such interactions should result in larger footprints that contain multiple TF recognition sites. Conversely, independent TF–DNA interaction events should yield compact and widely spaced footprints that contain single TF recognition sites. As such, the prevalence of cooperativity mediated by direct TF–TF interactions rather than by synergy of independent binding events should be reflected in the relative proportion of wide, multi-motif footprints compared to that of well-spaced single footprints. Larger footprints are overwhelmingly associated with two (or more) recognition sequences (Fig. 3a), but such footprints represent only 8% of the global footprint landscape. By contrast, 92% of footprints contain a single TF recognition site (Fig. 3b).

Because TFs can distort DNA upon engagement, TF spacing could be critical for establishing regulatory structures. To quantify global footprint spacing patterns, we first binned each DHS by its average accessibility across all biosamples (as footprint discovery depends on total DNase I cleavage; Extended Data Fig. 1b), and for each bin we computed the mean number of footprints present per element and their relative edge-to-edge spacing. The density of footprints within the most deeply sampled DHSs genome-wide plateaued at an average of 5.5 per 200 bp (Fig. 3c, top), which is in agreement with theoretical predictions of the number of human TFs required to destabilize a canonical nucleosome^26^ and to encode specificity^28^. Within DHSs, footprints exhibited average edge-to-edge spacing of about 21 bp (middle 50%, 12–35 bp) (Fig. 3c, bottom). Together, these results are compatible with the observed lack of evolutionary constraint on the spacing and orientation^29–33^ of TF motifs and strongly suggest that steady-state regulatory DNA accessibility is maintained chiefly by independent but synergistic TF binding modes (Fig. 3d).

## Cell-selective TF occupancy landscapes

**Extended Data Fig. 7:**
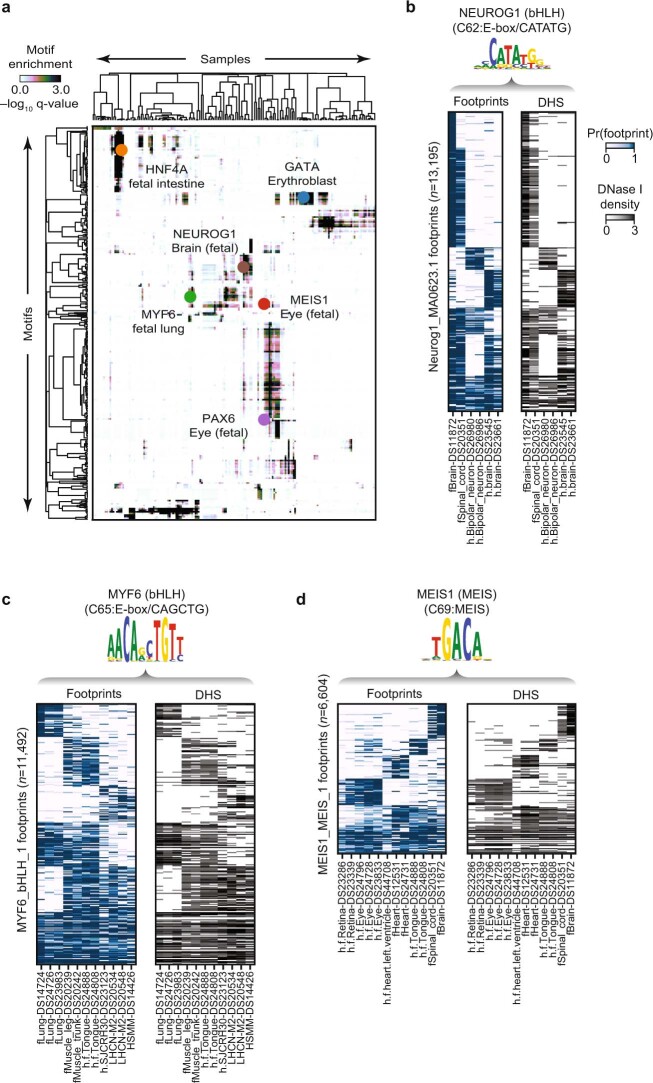
Cell-selective occupancy of TF recognition sequences. **a**, Hierarchically clustered heat map of TF recognition sequence enrichment (–log_10_ *q* values; Supplementary Methods) overlapping consensus footprints. Rows correspond to motifs and columns correspond to individual samples. **b**, Clustered heat maps of posterior probabilities for footprints (left) overlapping an E-box/CAGCTG (MYF6_bHLH_1 motif model) and their corresponding DNase I density (right) in each sample. Rows and columns are ordered using *K*-means (*k* = 6) and hierarchical clustering, respectively. **c**, **d**, Same as **b**, for footprints overlapping an E-box/CATATG (**c**, Neurog1_MA0623.1 motif model) or MEIS (**d**, MEIS1_MEIS_1 motif model) recognition sequence.

Footprint occupancy across all biosamples showed marked enrichment for the recognition sequences of key regulatory TFs in their cognate lineages (Extended Data Fig. 7a). In total, we identified 609 motif models that matched footprinted sequences (Supplementary Methods); these models encompassed 64 distinct archetypal TF recognition codes (Supplementary Table 2), representing virtually all major DNA-binding domain families. For degenerate motifs where the same sequence is recognized by many distinct TFs, we observed highly cell-selective occupancy patterns that could be decomposed into coherent groups that corresponded to cell type and function (Extended Data Fig. 7b–d). However, the cell-selective occupancy patterns of most individual TF footprints within DHSs mirrored the cell-selective actuation of their encompassing DHS (Extended Data Fig. 7b–d).

**Extended Data Fig. 8:**
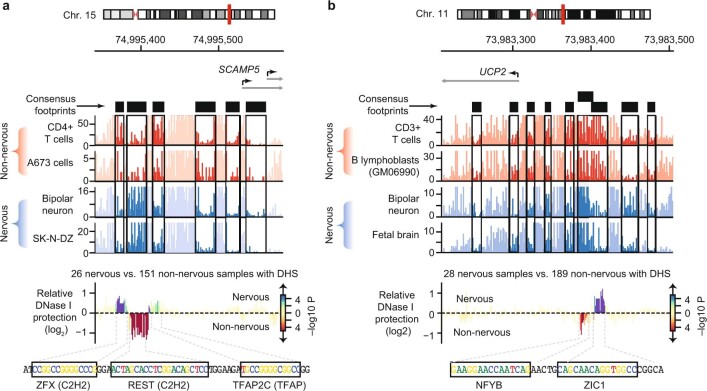
Comparative footprinting identifies cell-selective TF occupancy at nucleotide resolution. **a**, **b**, Comparative footprinting within the *SCAMP5* (**a**) and *UCP2* (**b**) promoters identifies footprints that are differentially occupied in nervous cell and tissue types. Top, DNase I cleavage in two exemplar nervous and non-nervous cell types. Bottom, mean differential per nucleotide cleavage (log_2_ observed/expected) between nervous system-derived (*SCAMP5*: *n =* 26; *UCP2*: *n =* 28 out of 31) and non-nervous samples (*SCAMP5*: *n =* 151; *UCP2*: *n =* 189 out of 212) in which region is DNase I hypersensitive (Supplementary Methods). The colour of each bar indicates the statistical significance (–log_10_ *p*) of the per-nucleotide differential test.

**Extended Data Fig. 9:**
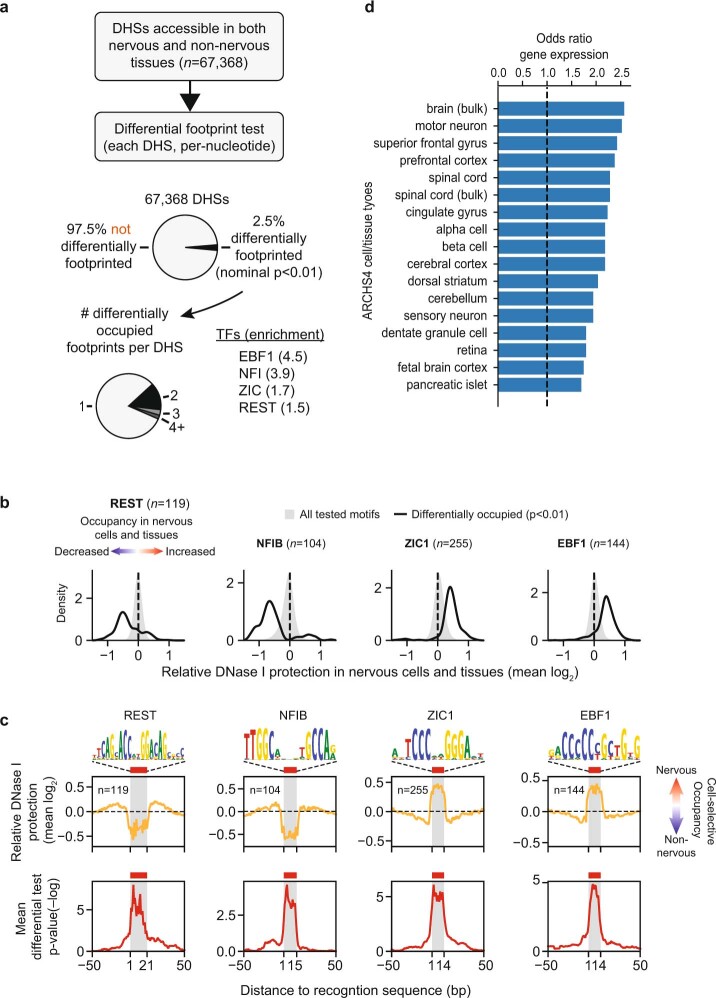
Differentially occupied nucleotides reflect aggregate DNase I cleavage profiles. **a**, Differential footprint testing within thousands of accessible DHSs between nervous and non-nervous related biosamples. The vast majority of tested DHSs encode a single TF binding topology. Top, percentage of the DHSs tested that containing one or more differentially occupied element. Bottom left, distribution of differentially footprinted elements per DHS. Bottom right, selected TF recognition sequences significantly enriched in differentially occupied footprints (binomial test *P* < 0.01). Indicated in parenthesis is the fold-enrichment vs expected (based on prevalence of footprinted motif in tested regions). **b**, Density histograms of relative footprint occupancy between nervous-system derived and non-nervous-system derived samples for the TF recognition sequences of REST, NFIB, ZIC1 and EBF1. Grey indicates distribution of all motif instances tested. Black indicates differentially footprinted. **c**, Per-nucleotide aggregate plots of the mean relative DNase I protection (top) and differential test *P* value (–log_10_, bottom) around differential occupied motifs. **d**, Cell- and tissue-specific expression of genes nearby differentially occupied REST footprints. Enrichment was performed using Enrichr^27^. Shown are cell and tissues with an adjusted Fisher exact test *P* value <0.01.

Given that most DHSs are shared across at least two cell types or states^13,34^, we queried how the pattern of footprints within a DHS (and hence its topology) differed with cellular context. Although differential TF occupancy can be discerned upon manual inspection^4^, systematic analysis has not been possible owing to the dominance of intrinsic DNase I cleavage propensities. To enable unbiased detection of differential footprint occupancy, we developed a statistical framework to test for differences in relative cleavage rates at individual nucleotides across many samples, analogous to methods developed for the identification of differentially expressed genes (Supplementary Methods). To estimate the proportion of differentially regulated footprints within DHSs of a given cell or tissue, we focused on the neural lineage, for which many biosamples were available. We compared footprint occupancy within DHSs that were broadly accessible in nervous-system-derived samples (*n* = 31) with that in non-nervous-system-derived samples (*n* = 212). We selected 67,368 DHSs that were highly accessible in at least 10 nervous- and non-nervous-derived samples, and for each DHS, performed a per-nucleotide differential test (Extended Data Figs. 8a, b, 9a). This analysis identified only a small proportion of DHSs (1,720 DHSs; 2.5%) as containing one or more differentially footprinted elements (Extended Data Fig. 9a). Most of these DHSs contained a single differentially regulated footprint, whereas a small fraction contained 2–4 differentially occupied elements (Extended Data Fig. 9a). Nonetheless, differentially occupied footprints were significantly enriched in recognition sites for known nervous system regulators such as REST, NFIB, ZIC1, and EBF1 (Extended Data Fig. 9a–c) and tissue-selective occupancy events paralleled the expression of nearby genes (in the case of REST occupancy) (Extended Data Fig. 9d).

Collectively, the above results indicate that the vast majority of regulatory DNA regions marked by DHSs encode a single structural topology that reflects a fixed pattern of footprint occupancy. Nonetheless, at a small minority of elements, DHSs provide a scaffold for cell-context-specific TF occupancy that is typically confined to one or a small number of footprinted elements.

## Functional DNA variants in TF footprints

Identifying genetic variants that are likely to affect regulatory function has remained challenging. Deep sequence coverage at DHSs enables de novo genotyping of regulatory variants and simultaneous characterization of their functional effect on local chromatin architecture by quantifying and comparing cleavage for each allele^2,4^. The 243 biosamples we analysed were derived from 147 individuals, and de novo genotyping (Supplementary Methods) revealed 3.76 million single-nucleotide variants (SNVs) within DHSs, of which 1,656,597 were heterozygous and had sufficient read depth (at least 35 overlapping reads) to accurately quantify allelic imbalance.

**Fig. 4:**
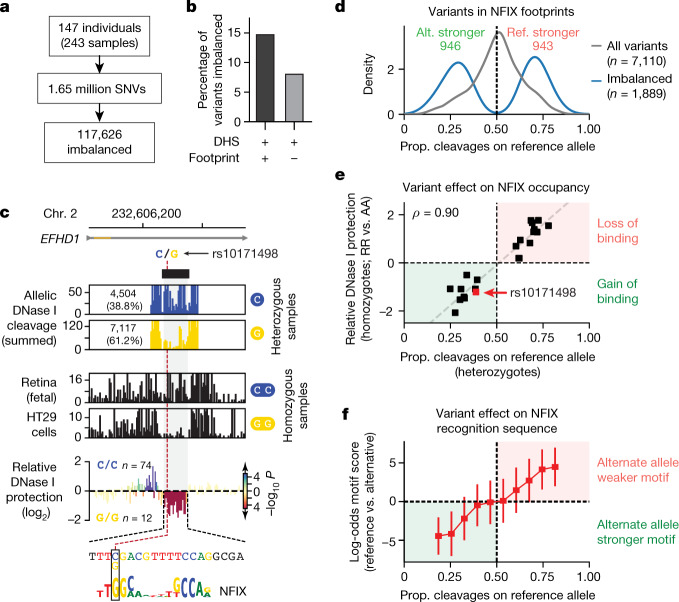
Functional genetic variation localizes in TF footprints. **a**, Allelic imbalance assessed at 1.65 million variants discovered from 147 unique individuals represented in 243 biosamples. **b**, Percentage of variants imbalanced in footprinted and non-footprinted segments of DHS peaks. **c**, Variant rs10171498 (C→G; C allele ancestral) creates a de novo NFIX footprint. Top, allelically resolved per-nucleotide DNase cleavage aggregated from 56 heterozygotes. Middle, DNase cleavage in two samples homozygous for reference or alternative alleles. Bottom, mean differential per-nucleotide cleavage (log_2_) between homozygous reference (*n* = 74) and alternative allele samples (*n* = 12). Colour indicates statistical significance (–log_10_ *P*) of per-nucleotide differential test (Methods). Variant and differentially footprinted nucleotides precisely colocalize at the NFIX element. **d**, Histogram of allelic ratios for variants that overlap the footprinted NFIX recognition sequence. Grey, all variants tested (*n* = 7,110). Blue, significantly imbalanced variants (*n* = 1,889). Prop., proportion. **e**, Scatter plot of allelic imbalance in heterozygous individuals (*x*-axis) against relative difference in footprint depth between homozygous individuals at variants overlapping an NFIX footprint. Each point shows an individual SNV within the footprinted NFIX binding site that is both imbalanced (*q* < 0.2) in heterozygotes and differentially footprinted (nominal *P* < 0.05) in homozygotes. Grey line, fitted linear model. **f**, Allelic imbalance versus predicted energetic effects of variants within NFIX footprints. Shown is median log-odds score (reference versus alternate allele) of all tested variants within footprinted motifs binned by allelic ratio. Error bars show 5th and 95th percentiles of log-odds motif scores in each bin.

**Extended Data Fig. 10:**
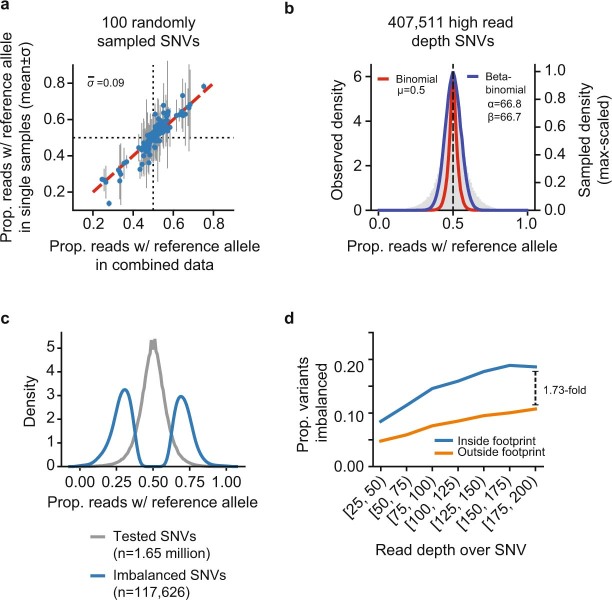
Detection of chromatin-altering variants. **a**, Scatter plot of allelic ratios at 100 randomly selected high-confidence SNVs (Supplementary Methods) computed after aggregating reads from different samples (*x*-axis) against the distribution of allelic ratios at the same SNVs in each sample (*y*-axis; mean ± s.d.). The average s.d. indicated in the top left corner was used to tune the parameters of a beta-binomial distribution. **b**, Simulation of allelic ratios from the observed total read depth at high-confidence SNVs assuming a binomial distribution (*P* = 0.5) or a beta-binomial distribution. Grey indicates the observed allelic ratios at the same variants. **c**, Density histogram of allelic ratios for all tested SNVs (grey line) and significantly imbalanced SNVs (blue line). **d**, Proportion of SNVs imbalanced with respect to read depth for variants within (blue) or outside (orange) consensus footprints (posterior probability >0.99).

Across individuals, we conservatively identified 117,626 chromatin-altering variants (CAVs) that altered DNA accessibility on individual alleles (median 2.4-fold imbalance) (Fig. 4a, Extended Data Fig. 10a–c, Supplementary Methods). Within DHSs, CAVs were markedly enriched in core consensus footprints, even after controlling for the increased detection power (that is, sequencing depth) within this compartment (Fig. 4b, Extended Data Fig. 10d).

**Extended Data Fig. 11:**
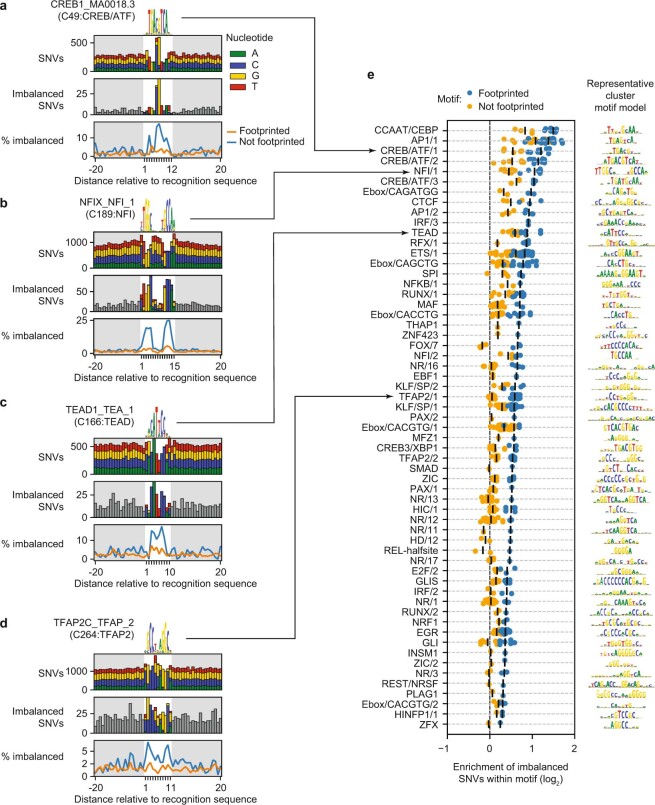
Enrichment of imbalanced variants within footprinted TF recognition sequences. **a**–**d**, Distribution of SNVs around the recognition sequences for CREB/ATF, NFI, TEAD and TFAP2 TFs. For each TF, shown are the total SNVs tested for imbalance (top), imbalanced variants (middle), and the proportion of variants imbalanced stratified overlap with a consensus footprint. **e**, log_2_ enrichment of imbalanced variants residing within TF recognition sequences relative to non-imbalanced SNVs for both variants within footprinted (blue) and non-footprinted motifs (orange). Motifs are grouped into clusters, where each point represents an individual motif model (Extended Data Fig. 4, Supplementary Table 2 and Supplementary Methods). Black bars indicate mean enrichment across all motifs in each cluster and footprint overlap. Only motifs with significant (*q* < 0.05) enrichment of imbalanced SNVs with a footprinted recognition sequence are shown.

**Extended Data Fig. 12:**
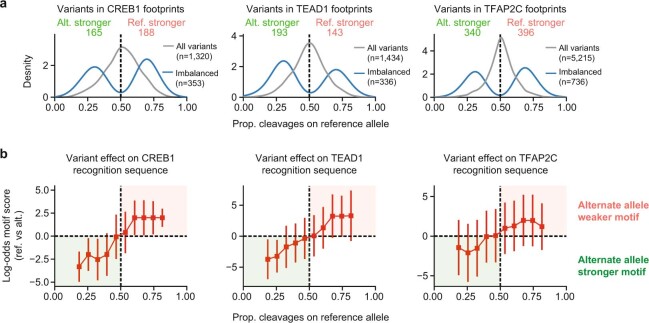
Allelic imbalance parallels the predicted energetic effect of genetic variation. **a**, Histogram of allelic ratios for variants overlapping footprinted CREB1 (CREB/ATF), TEAD1 (TEAD) and TFAP2C (TFAP2) recognition sequence. Grey line, all variants tested for imbalance. Blue line, all variants significantly imbalanced. **b**, Median log-odds score (reference versus alternate allele) of all tested variants within footprinted motifs binned by allelic ratio. Error bars show 5th and 95th percentiles of log-odds motif scores in each bin.

In protein-coding regions, most functional genetic variation is expected to be deleterious, with rare gain-of-function alleles^35^. Protein–DNA recognition interfaces are likewise presumed to be susceptible to disruption at critical nucleotides, predisposing to loss-of-function alleles^36^. Notably, we found that CAVs were nearly evenly partitioned between loss-of-function (disruption of binding) and gain-of-function (increased or de novo binding) alleles (Fig. 4c, d, Extended Data Fig. 10c). Homozygosity for either the reference or alternative allele paralleled results from heterozygotes and further revealed that structural changes due to TF occupancy were precisely confined to the DNA sequence recognition interface (Fig. 4c, bottom). In many cases, SNVs that were detected in both heterozygous and homozygous configurations showed strong agreement between allelic ratios and relative footprint strength (Fig. 4e; Spearman’s *ρ* = 0.9, *P* < 10^−5^). Variants within footprinted motifs were markedly enriched for imbalance when compared to non-footprinted motifs; were localized to high-information-content positions within the recognition interface (Fig. 4c, bottom, Extended Data Fig. 11); and paralleled the predicted energetic effect of the variant on the TF binding site (Fig. 4f, Extended Data Fig. 12), thus providing a direct quantitative readout of the effects of functional variation on TF occupancy.

## TFs occupy hypermutable DNA

**Fig. 5:**
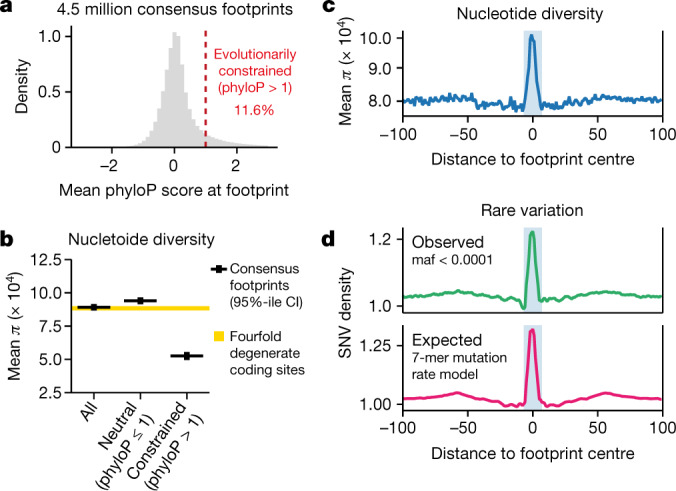
Footprints are highly polymorphic in human populations. **a**, Histogram of mean phyloP scores within consensus footprints. Dashed red line, phyloP = 1. **b**, Nucleotide diversity (*π*) within footprints (mean ± 95% confidence interval of mean). Yellow bar, 95% confidence interval of mean *π* computed at fourfold degenerate coding sites. **c**, Mean nucleotide diversity versus distance from consensus footprints. Shaded bar represents average footprint width (14 bp). **d**, Density of observed and expected rare variation within footprints. Top, variants with minor allele frequencies (maf) <0.0001. Bottom, expected rare variation computed using 7-mer sequence context mutation rate model^42^.

We next sought to characterize the patterns of human genetic variation within regulatory DNA with high precision. Only a small fraction (11.6%) of individual footprints showed evidence of evolutionary constraint (phyloP score >1), consistent with purifying selection, whereas the vast majority appeared to be evolving neutrally (Fig. 5a). To quantify the relationship between evolutionary constraint and genetic variation in human populations, we calculated mean nucleotide diversity (*π*) within consensus genomic footprints by using more than 400 million single-nucleotide variants detected by whole-genome sequencing of over 65,000 individuals under the TOPMED project^37^ (Fig. 5b, Supplementary Methods). Canonically, reduced levels of *π* reflect the elimination of deleterious alleles from the population by natural selection, and hence are indicative of recent functional constraint. Consistent with prior observations^36^, we found that mean *π* within footprints approximated that of fourfold degenerate sites within protein-coding regions, which are assumed to be evolving neutrally or under relaxed selection. Stratification of footprints by the level of evolutionary constraint (phyloP score >1) revealed marked differences in genetic diversity, with significantly reduced levels of *π* within highly evolutionarily constrained footprints and increased *π* in non-constrained footprints (*P* < 0.0001; two-sample bootstrap *t*-test).

The density of sampled variation enabled nucleotide-resolution analysis of nucleotide diversity at footprinted and non-footprinted bases within DHSs. Unexpectedly, we found a marked increase in nucleotide diversity centred precisely within the core of footprints (Fig. 5c), revealing that these elements as a class—but not intervening non-footprinted segments of DHSs—are highly polymorphic in human populations. This result eclipses prior global analyses indicating that TF occupancy sites are generally not under substantial purifying selection^4,36^ both in the magnitude of the observed effect and in its nucleotide-precise localization within the footprint core.

Focally increased genetic diversity within footprints suggested that the nucleotides that encode these elements may have an increased mutational load when compared with immediately adjacent sequences. To explore this possibility, we focused on variants with extremely low allele frequencies in human populations (minor allele frequency less than 10^−4^); such variants are assumed to result from de novo germline (that is, non-segregating) mutation and are often used as a surrogate for mutation rate in humans. We found that the distribution of extremely rare variants within and around footprints mirrored that of nucleotide diversity, compatible with increased mutation rate within footprints (Fig. 5d, top). TFs have been hypothesized to potentiate de novo mutation by focally inhibiting access by the DNA repair machinery^38,39^. Nucleotide context is also known to have a substantial role in genome mutation^40^, and this can be accurately modelled across a wide range of nucleotide combinations^41,42^. To differentiate these possible causes of increased mutation, we used a 7-mer context mutation rate model^42^ (Supplementary Methods) to predict mutation density within footprints. This model nearly completely recapitulated the observed density of human SNVs within footprints (Fig. 5d, bottom), indicating that footprint mutational load derives chiefly from local sequence composition and not from a repair-mediated process.

**Extended Data Fig. 13:**
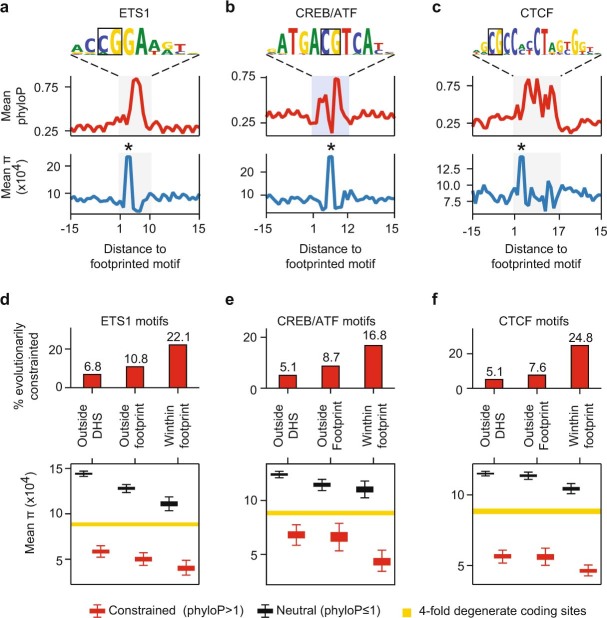
Nucleotide-resolved patterns of genetic variation within TF binding sites. **a**–**c**, Per-nucleotide profiles of phyloP scores (top) and human nucleotide diversity (*π*) (bottom) within footprinted motifs for ETS1 (**a**), JDP2 (**b**), and CTCF (**c**) motifs. Black box in the motif consensus logo annotates CpG dinucleotides. Asterisk indicates the position of the CpG dinucleotide in the profiles below. **d**–**f**, Ancient and recent constraints at the TF recognition sequences with respect to proximity to DHSs and consensus footprints. TF recognition sequences are grouped by those residing within ±5 kb of DHS peaks but not inside (outside DHS), inside DHSs but not a footprint (outside footprint) and those overlapping a consensus footprint. Top, percentage of TF recognition sequence under elevated evolutionary constraint (mean phyloP score >1) in each group. Bottom, mean nucleotide diversity within the footprinted motifs additionally stratified by evolutionary constraint. Boxes indicate median and IQR of enrichments from 1,000 bootstrap samples. Whiskers, 5th and 95th percentile.

Mutational mechanisms have been linked to the observed widespread turnover of TF recognition sites^43,44^. Of note, many TFs favour the recognition of dinucleotide combinations such as CpGs that are intrinsically hypermutable or of dinucleotides that result from CpG deamination^43,44^. We examined the nucleotide-resolved patterns of both evolutionary conservation and genetic variation at footprinted motifs for structurally distinct TFs with CpG dinucleotides in their core recognition sequence (ETS1, JDP2 and CTCF). For each motif, conservation and nucleotide diversity were reciprocal, and mutations at CpG dinucleotides appeared to be the key drivers of generic diversity (Extended Data Fig. 13a–c).

Because increased polymorphism within TF footprints is attributable to variability in mutation rates resulting from sequence context, it remains unclear to what extent purifying selection is acting on TF occupancy. To quantify this, we compared footprinted motifs to non-footprinted elements (both within and outside DHS), reasoning that the latter should represent neutrally evolving, non-functional sites, but should be subjected to similar mutational forces owing to proximity. Consistent with this, footprinted motifs were markedly more evolutionarily constrained (approximately threefold to fivefold) than non-footprinted motifs (Extended Data Fig. 13a–c, top). For each TF, we found that footprinted motifs had lower aggregate nucleotide diversity than non-footprinted elements, yet these differences were largely overshadowed by differences between evolutionarily constrained and unconstrained motifs (Extended Data Fig. 13d–f, red and black boxes, respectively). These results indicate that while a core set of binding sites appears to be under substantial constraint (on a par with protein-coding regions), the vast majority of footprints appear to be under very weak selective constraint. Notably, for each of the three aforementioned TFs, mutations that occurred within their footprinted motifs preferentially modulated allelic imbalance in chromatin accessibility, linking natural variation to functional variation (Extended Data Fig. 11). Thus, hypermutation within genomic footprints appears to have a key evolutionary role by favouring variability in TF occupancy and hence natural variation in gene regulation.

## GWAS variants localize within TF footprints

**Fig. 6:**
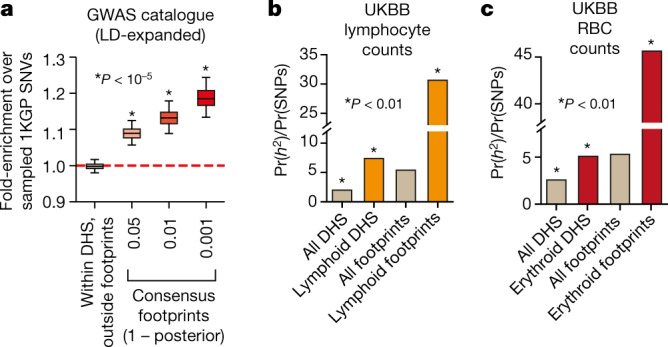
Trait-associated variation is concentrated in consensus footprints. **a**, Enrichment of GWAS variants within or outside consensus footprints versus randomly sampled 1,000 Genomes Project (1KGP) variants, after expanding both with variants in perfect LD (*r*^2^ = 1.0, central European population). Centre lines, median; boxes, interquartile range (IQR); whiskers, 5th and 95th percentile (of enrichments from 1,000 sampling iterations). Statistical significance determined by a normal distribution fitted to sampled data (Supplementary Methods). **b**, **c**, Enrichment of SNP-based trait heritability using LD-score regression for UK BioBank (UKBB) GWAS on lymphocyte count (**b**) and red blood cell count (**c**). Pr(*h*^2^), proportion of trait narrow-sense heritability explained by SNPs overlapping DHSs or footprints. Pr(SNPs), proportion of total SNPs within each annotation. Asterisk, enrichment *P* < 0.01.

Given the above, genetic variation within footprints should, in principle, be a key contributor to phenotypic variation. We therefore next resolved the large set of variants that are strongly associated (nominal *P* < 5 × 10^−8^) with diverse diseases and phenotypic traits from the NHGRI/EBI genome-wide association study (GWAS) catalogue^45^ to consensus genomic footprints. To account for the baseline increase in genetic variation present within the genomic footprints described above, we performed exhaustive (1,000×) sampling of matched variants (by minor allele frequency, linkage-disequilibrium (LD) structure, and distance to the nearest gene) from the 1,000 Genome Project^46^ (Supplementary Methods). In addition, we expanded both GWAS catalogue and matched sampled variants to include variants that were in perfect LD (*r*^2^ = 1). Within DHSs, aggregated GWAS catalogue SNPs were enriched within footprints but not non-footprinted subregions, and the former increased monotonically with footprint strength (Fig. 6a).

To gain a more accurate view of the enrichment of trait-associated variants in footprints, we compared the SNP-based trait heritability of individual traits^47,48^. Using summary statistic data from individual GWAS studies from the UK BioBank, we applied partitioned LD-score regression to compute the relative heritability contribution of variants within all DHSs and footprints collectively versus that of DHSs and footprints from the expected cognate cell type for a given trait (Fig. 6b, c). We found striking enrichment of variants that account for trait heritability in footprints generally (more than fivefold) and most prominently in footprints from the cognate cell type (up to approximately 45-fold) (Fig. 6b, c). We thus conclude that the genetic signals from disease- and trait-associated variants within DHSs emanate from TF footprints, and that variants within footprints are major contributors to trait heritability.

## Discussion

We have described the highest-resolution view to date of regulatory factor occupancy patterns on the human genome, measured across an expansive range of cell and tissue contexts sampled from more than 140 genotype backgrounds. The scale and breadth of the data have enabled delineation of a reference set of about 4.5 million genomic sequence elements that form the building blocks of regulatory DNA and collectively define nucleotides that are crucial for genome regulation and function. While expansive, this catalogue is nonetheless not comprehensive owing to incomplete sampling of human cell types and states, and non-exhaustive sequencing of individual DNase-seq libraries. We note further that the algorithms we have applied, while incorporating considerable advances over prior efforts, nonetheless incompletely exploit the richness and subtleties of the measured cleavage landscape.

Assigning individual TFs to individual footprints presents many challenges. Here, we applied a de novo approach in which TFs were assigned to footprints post hoc via overlap with their cognate recognition sequences. A complicating factor is that many functionally distinct TFs use similar recognition sequences, leading to potential ambiguous assignment of TFs to individual footprints. In addition, co-expressed TFs with similar recognition sequences may alternatively occupy the same element^49^. Because DNase I cleavage patterns encode rich information about the topology and binding modes of individual factors (Fig. 2, Extended Data Fig. 6), incorporating this information into future approaches should greatly increase the fidelity of TF–footprint assignments.

Collectively, the consensus footprint index now provides a ready and extensible nucleotide-precise reference for diverse analyses, particularly those involving genetic variation. The preferential localization of disease- and trait-associated variation within regulatory DNA has heretofore been described in terms of entire regulatory regions demarcated by DHSs or clusters thereof. Our results now show that genetic association and heritability signals from regulatory DNA overwhelmingly emanate from consensus TF footprints, which should greatly facilitate the connection of disease- and trait-associated genetic variation with genome function.

Perhaps most notably, we report that human genetic variation is itself concentrated within TF footprints, owing apparently to a combination of mutation propensity and the evolved sequence recognition repertoire of human TFs, which favours hypermutable nucleotide combinations (for example, CpG dinucleotides). Given that human and mouse TFs share the large majority of their recognition landscapes, the concentration of variation within TF occupancy sites is likely to have had a considerable role in shaping mammalian regulation^50^; furthermore, this finding suggests that genomes are heavily primed for regulatory evolution, providing a possible underlying mechanism for facilitated phenotypic evolution^51^.

### Reporting summary

Further information on research design is available in the Nature Research Reporting Summary linked to this paper.

## Online content

Any methods, additional references, Nature Research reporting summaries, source data, extended data, supplementary information, acknowledgements, peer review information; details of author contributions and competing interests; and statements of data and code availability are available at 10.1038/s41586-020-2528-x.

## Supplementary information

Supplementary Methods and References.

Reporting Summary

Supplementary Table 1 | Overview and summary of DGF data Biosample metadata corresponding to all dataset utilized in this study.

Supplementary Table 2 | Clustering of TF recognition sequence models Assignment of individual motifs models to non-redundant motif clusters.

Supplementary Table 3 | ChIP-seq data used for TF occupancy classification Description and overview of ENCODE ChIP-seq and DNase I data used for classification of TF occupancy.

## Data Availability

All raw and processed DNase-seq data are available through the ENCODE portal (http://www.encodeproject.org/) under accessions in Supplementary Table 1. Footprints and their metadata are available at http://vierstra.org/resources/dgf or 10.5281/zenodo.3603548 (Zenodo). A track hub to visualize data in the UCSC Genome Browser is hosted at https://resources.altius.org/~jvierstra/projects/footprinting.2020/hub.txt. Protein structures for CTCF (Fig. 2a) and PAX6 (Extended Data Fig. 6a) were downloaded from Protein Data Bank (https://www.rcsb.org) (PDB IDs: 5YEF, 5YEL, and 6PAX).

## Code availability

Footprint detection software is available at http://www.github.com/jvierstra/footprint-tools. All code for analyses herein is available upon request.
