# Supplementary material for: Global reference mapping of human transcription factor footprints
Source: Nature. 2020 Jul 29;583(7818):729–36. doi: 10.1038/s41586-020-2528-x (PMC7410829; doi:10.1038/s41586-020-2528-x)
Supplement: Supplementary file 1 — Supplementary Methods and References. [file 41586_2020_2528_MOESM1_ESM.pdf]

nature

https://doi.org/10.1038/s41586-020-2528-x

Supplementary information

Global reference mapping of human transcription factor footprints

In the format provided by the authors and unedited

Jeff Vierstra✉, John Lazar, Richard Sandstrom, Jessica Halow, Kristen Lee, Daniel Bates, Morgan Diegel, Douglas Dunn, Fidencio Neri, Eric Haugen, Eric Rynes, Alex Reynolds, Jemma Nelson, Audra Johnson, Mark Frerker, Michael Buckley, Rajinder Kaul, Wouter Meuleman & John A. Stamatoyannopoulos✉

Table of contents

Supplementary Methods **2**
Digital genomic footprinting data 2
DNase I sequence preference model 2
Computing expected per-nucleotide cleavage rates 2
Derivation of cleavage dispersion model 3
Per-nucleotide cleavage testing 3
Bayesian modeling 4
Derivation of consensus footprints 5
Testing for differential DNase I cleavage 5
Evolutionary conservation 6
Transcription factor binding site predictions 6
Clustering TF recognition sequences by similarity 6
Assignment of TF recognition sequences to consensus footprints 6
Enrichment of TF recognition sequences within consensus footprints 6
Classification of ChIP-seq data 7
Co-crystal structural modeling 7
Genotyping from DNase I sequence reads 7
Detecting allelic imbalance 7
Enrichment of imbalanced variants within TF recognition sequences 8
Energetic effects of variation on TF recognition sequences 8
Human genetic variation 9
Deriving expected density of rare variants 9
GWAS variation 9
Stratified LD-score regression analysis 10

Supplementary References **10**

Supplementary Methods

Digital genomic footprinting data

The digital genomic footprinting datasets used in this study were released as part of the ENCODE[1] and Roadmap Epigenomics Consortia[2]. DNase I digestion, purification of small double-hit fragments and sequencing library preparation was performed as in ref. 3. Sequencing was performed on the HiSeq platform (Illumina). Raw sequencing reads were trimmed to remove adapter sequences and aligned to the human genome (hg38/GRCh38) using bwa[4] version 0.7.12) with the following parameters: "`-Y -l 32 -n 0.04`" and "`-n 10 -a 750`" for alignment and mate-pairing (`aln` and `sampe`, respectively). DHS peaks (*i.e.*, hotspots) were determined using (hotspot2; http://github.com/Altius/hotspot2).

DNase I sequence preference model

A sequence model of DNase I preference was constructed as in refs. 5,6. Briefly, deproteinated genomic DNA derived from IMR90 (fetal lung fibroblasts) cells was digested with bovine DNase I (Sigma-Aldrich). DNase I-released fragments isolated, processed and sequenced as described above. For each uniquely mapping sequencing tag, the 5' alignment position, i, was used to extract the DNA sequence hexamer covering positions $[i-3, i+2]$ with respect to the strand of the alignment. The total amount of each hexamer was normalized by the total number of hexamers (considering both strands). We excluded positions in the that genome that cannot be mapping uniquely using within the 36-bp reads to control for confounding effects that short sequence mappability may have on our empirically derived hexamer cleavage model (UCSC Genome Browser "Mappability Track/CRG Alignability 36bp[7,8]).

Computing expected per-nucleotide cleavage rates

Expected cleavage rates are computed by redistributing cleavage counts to the DNase I sequence preference model described above. First, the total cleavages (*w*) within a ±5 bp of each base (11 bp window) genome-wide is calculated such that:

$$w_i = \sum_{j=i-5}^{i+5} n_j \qquad \textit{Eq. 1}$$

From these values a sliding trimmed mean is computed (removing the top and bottom 1%) in ±50 bp intervals (101 bp window) which represents an estimate of total expected cleavages within ±5 bp of each nucleotide.

$$w_i^s = \overline{(w_{i-50}, \dots, w_{i+50})}_{0.01} \qquad \textit{Eq. 2}$$

Next, the underlying hexamer sequence for each nucleotide, *i*, is used to create any array of relative sequence preference across the genome (a). These relative preference values are then normalized in ±5 bp sliding windows (as above).

$$p_i = \frac{a_i}{\sum_{j=i-5}^{i+5} a_j} \qquad \textit{Eq. 3}$$

Finally, the expected cleavages for each nucleotide (n_i') is determined by multiplying the expected cleavages in a ±5 bp window to its normalized relative preference value at the same position.

$$n_i' = p_i w_i^s \qquad \textit{Eq. 4}$$

An expected count is derived independently for each strand and are combined to generate a final aggregated expected count.

Derivation of cleavage dispersion model

Statistical detection of footprints uses a properly fit per-nucleotide cleavage dispersion model from which to test whether observed cleavage rates significantly deviate from the expected. A dispersion model is created independently from the observed data in three steps:

1. **Compute expected cleavage rates.** Expected cleavage rates are generated from the observed cleavages rates (as above), except without windowed smoothing (*i.e.*, $n_i' = p_i w_i$). Windowed smoothing was omitted when training the dispersion model in order to estimate the cleavage variance driven exclusively by local sequence effects (±10 bp).

2. **Fit parameters for statistical distribution.** For each predicted cleavage rate all of the observed rates at their corresponding sites are collected and the parameters of a negative binomial distribution are fitted to the observed rates by maximum likelihood estimation

3. **Local smoothing and extrapolation of model parameters.** As the predicted cleavage rate increases the number of observations decreases (due to a preponderance of low cleavage rates). To reduce the noise in fitting negative binomial distributions to each predicted cleavage rate and to estimate the NB parameters in the case of insufficient data, a piecewise linear regression is performed to determine the parameters with respect to the predicted cleavage rate. The resulting regression coefficients are used to compute the negative binomial parameters in all subsequent analysis.

Per-nucleotide cleavage testing

The significance of cleavage rate deviation at a nucleotide *i* is determined by comparing its observed cleavages to the cleavage rate expected at an unoccupied nucleotide. We generated the expected cleavage rates genome-wide as described above using windowed smoothing ($n_i' = p_i w_i^s$). We compute per-nucleotide deviation *p*-values from the lower-tail of a negative binomial distribution parameterized by μ and *r* from the dispersion at the expected cleavage rate. The lower tail of the negative binomial is computed using the incomplete beta function. A footprint is defined by short stretches of nucleotides which display relative protection from cleavage due to protein engagement. In the null case (no footprint), the cleavage rates at

adjacent nucleotides are independent (**Extended Data Fig. 1d**). As such, we test for joint local deviation of cleavage rates by combining adjacent per-nucleotide *p*-values in a local window (±3 bp; 7 bp total) using Stouffer's *Z*-score method.

Calibration of *p*-values to account for multiple testing is performed empirically by sampling cleavage counts from the observed variance in the dispersion model. Specifically, each the cleavage count for each nucleotide is resampled from the expected distribution per the dispersion model. Sampled nucleotides are then processed identical to the observed cleavage rates, such that per-nucleotide and windowed *p*-values are computed. This sampling procedure was performed 1,000 times independently and the *p*-values were aggregated to generate a reference null distribution. The observed *p*-values are sorted and ranked against the null distribution to yield an empirical false discovery rate.

Bayesian modeling

We formulated an Empirical Bayes framework that computes posterior *p*-values after considering all observed data. This method is used to generate a list of high-confidence reference footprints across hundreds of samples. The posterior probability (*p*) of a footprint at individual nucleotide is:

$$p(\theta_+|X,Y) = \frac{P(\theta_+)P(X,Y|\theta_+)}{P(\theta_+)P(X,Y|\theta_+) + \left(1 - P(\theta_+)\right)P(X,Y|\theta_-)} \quad \textit{Eq. 5}$$

where, X corresponds to the observed cleavage rates and Y represents the expected cleavage rates. The footprint prior, $P(\theta_+)$, is the number of datasets that a nucleotide is found within a footprint (FDR<0.05) divided by the number datasets in which that nucleotide resides within a DNase I hypersensitive site (as defined by hotspot2). The likelihood corresponding an *unoccupied nucleotide*, $P(X,Y|\theta_-)$, is the product of individual negative binomial probabilities corresponding to dispersion model parameterized by the expected cleavage rate.

$$P(X,Y|\theta_-) = \prod_{j=i-3}^{i+3} \binom{x_j + r_j - 1}{x_j} \left(1 - p_j\right)^{r_j} p_j^{x_j} \quad \textit{Eq. 6}$$

$$\theta_- = \Phi_{NB}(y_i) = (p_i, r_i) \quad \textit{Eq. 7}$$

Here, $\Phi_{NB}(y_i)$ is the function that returns the parameters (p_i, r_i) of the fitted negative binomial at the expected cleavage rate y_i (see "*Local smoothing and extrapolation of model parameters*" above). The likelihood function corresponding an *occupied nucleotide*, $P(X,Y|\theta_+)$, is determined similar to the unoccupied case after scaling the expected cleavage rate by the expected depletion of cleavage at occupied nucleotides:

$$\theta_+ = \Phi_{NB}(y_i\lambda_i) = (p_i^+, r_i^+) \quad \textit{Eq. 8}$$

where λ_i is the expected depletion of cleavage at an occupied nucleotide. We determine λ_i by considering all datasets with an FDR 5% footprint at position j. First, for each dataset we fit a Beta distribution to the ratio of observed over expected cleavages (depletion ratio) at all FDR 5% footprints identified within individual datasets (capping the ratio values at 1.0). Then, for each nucleotide we re-estimate the depletion ratio by updating the Beta distribution ($\alpha' = \alpha + x; \beta' = \beta + (y_i - x_i)$). These updated parameters are used to generate maximum *a posteriori* (MAP) estimates of the depletion ratio (μ_{MAP}) and expected variation of this ratio (σ^2_{MAP}) at each nucleotide. A per-nucleotide footprint depletion estimate is then finally calculated from the average of the MAP mean estimates weighted by the inverse of the MAP standard deviation considering all datasets with an identified footprint at that nucleotide.

Derivation of consensus footprints

We derived an index of footprints in the human genome by considering the total collection of all per-dataset footprints called at posterior probability > 0.99. Briefly, for each genomic locus, we aligned the location and dispersion of footprints across datasets to delineate consensus coordinates supported by at least 50% of all footprint-contributing datasets. This approach is identical to the one used to delineate consensus DNase I hypersensitive sites (DHSs) as used in the accompanying manuscript by Meuleman *et al.*[9] We note that for some analysis, consensus footprints were established at both lesser and more stringent thresholds (*e.g.*, **Fig. 6a)**.

Testing for differential DNase I cleavage

We created a test whether modeling distributions of cleavage rates as two groups is more likely than modelling a data as a single group. The test models the $\log_2$ transformed observed vs. expected ratios as normal distributions with known mean but with uncertain variance estimates. As such, we model the cleavage rate variation using a Bayesian approach. We estimate prior hyperparameters on the nucleotide cleavage ratio variance using a scaled-inverse $\chi 2$ distribution from all positions within a DHS. The scaled-inverse $\chi 2$ distribution is a conjugate prior to the normal distribution in which the Student's t results as the predictive posterior distribution of the observed vs. expected cleavage rates. The likelihood of the data is the product of the posterior probability distribution of the cleavage ratio with the likelihood the observed (X) and expected (Y) cleavage rates (see Eq. 6):

$$\mathcal{L}(\mu, v', \sigma^{2\prime} | X, Y) = \sum_{i=1}^{n} \int f(x_i, y_i | \theta)\, t_{v'}(\theta | \mu, \sigma^{2\prime}) d\theta$$

$$v' = v_o + n \qquad \textit{Eq. 9}$$

$$\sigma^{2\prime} = \frac{v_o \sigma_0^2 + \sum_{i=1}^{n} (\phi_i - \mu)^2}{v_o + n}$$

where v_o and σ_0^2 are the (hyper)parameters of the fitted scaled-inverse $\chi2$ distribution, μ is the mean of the $\log_2$ transformed observed vs. expected cleavage ratios (ϕ) and n is the number of samples. The likelihood of the data is computed considering two groups or as a single group using numerical approximation. A log-likelihood ratio is computed as $LLR = \ell_{AB} - (\ell_A + \ell_B)$. A likelihood ratio test (χ^2; 3 degrees of freedom) is used to test for statistical differences between the models (two groups vs. one group) and hence differential footprints. We combined adjacent per-nucleotide *p*-values in a local window (±3 bp; 7 bp total) using Stouffer's *Z*-score method.

Evolutionary conservation

The per-nucleotide phyloP[10] 100-way conservation track was downloaded from the UCSC Genome Browser corresponding to human genome build hg38/GRCh38 used for all analyses.

Transcription factor binding site predictions

TF recognition sequence models were obtained from a large scale SELEX[11], JASPAR (2018)[12], and HOCOMOCO (version 11)[13] and scanned genome-wide using the software package MOODS (v1.9.3)[14] with the following parameters: `"--p-value 1e-4 --lo-bg 0.2977 0.2023 0.2023 0.2977"`

Clustering TF recognition sequences by similarity

To systematically collapse redundant motifs by similarity we used an approach similar to Maurano et al.[15]. Briefly, we used TOMTOM[16] to compute the distances between all motif pairs (2,174 motif models). We then performed hierarchical clustering using Pearson correlation as the distance metric and complete linkage. The tree was cut at height 0.7. For each of the 286 clusters, we then randomly selected a seed motif model to which we aligned all other motifs within cluster (both position and orientation).

Assignment of TF recognition sequences to consensus footprints

To assign TF recognition sequences to consensus footprints, we collapsed genome-wide transcription factor binding site predictions of all TF models by translating the coordinates and orientation of motif match into relative to its assigned cluster, removing redundant assignments of the same motif cluster at identical genomic coordinates. We then overlapped these collapsed motifs with consensus footprints with BEDOPS (v2.4.39)[17], requiring that either 90% of motif is overlapped by footprint or vice-versa (*e.g.*, `"bedmap --fraction-either 0.9 --echo –echo-map-id <footprints> <motifs>"`)

For all other analyses, all genome-wide TF recognition sequence matches were overlapped (≥3bp) with reference footprints (posterior probability>0.99) (*e.g.*, `"bedmap –bp-ovr 3 --echo –echo-map-id <footprints> <motifs>"`).

Enrichment of TF recognition sequences within consensus footprints

For each biosample, we computed the fraction of occupied consensus footprints (posterior footprint probability > 0.99) overlapping matches to each motif model. To compute cell-selective

enrichment, we calculated the mean and standard deviation of the fractional occupancy of each motif across all biosamples after removing outliers (>3.5 times the median absolute deviation) We used these values to parametrize a normal distribution and derive a upper-tail p-value for each biosample. *P*-values were adjusted using the Benjamini-Hochberg method to control for multiple testing.

Classification of ChIP-seq data

ChIP-seq data ("optimal IDR thresholded peaks") was downloaded from the ENCODE Project portal website (http://www.encodeproject.org) (**Supplementary Table 3**). We filtered selected matching DNase I/ChIP dataset combination requiring that (i) 80% of ChIP-seq peaks overlap DNase I hotspots and (ii) >50% of ChIP-seq peaks overlapped a recognition sequence. For each matched DNase I/ChIP-seq dataset pair, we annotated all motifs within DHS hotspots by overlap with a ChIP peak (±100bp) and footprint posterior probability. For peaks with more than one motif match, we selected the motif closest to the DHS summit and excluded the remaining motifs from downstream analysis.

Co-crystal structural modeling

To visualize DNase I cleavage with respect to the physical CTCF physical structure, we obtained protein-DNA co-crystals corresponding to the ZFs 2-8 and 6-11 (PDB 5YEF and 5YEL)[18] and assembled them together *in silico* to generate a full-length structural representation of the DNA-binding domain interface. For PAX6, we obtained the TF:DNA co-crystal (PDB:6PAX[19]) and computationally predicted and aligned the structure of an extended fragment of DNA using Web 3DNA 2.0 (http://web.x3dna.org)[20] (sequence: `GCTCCTCTTAAGCATTTTCACGCATGAGTGCACAGACCTTAAGA)`. Assembly, analysis and visualization of structural data was performed using PyMOL (v2.3.2)[21].

Genotyping from DNase I sequence reads

Genotype information was ascertained directly from the DNase I data using a standard genotyping pipeline. First genotypes from individual datasets were called directly from the BAM files generated for digital genomic footprinting using bcftools (version 1.9) commands `mpileup` with the following parameters: `"-Q 20 -d 1000 -I -D -a FORMAT/DP,FORMAT/AD"` and `call` with the parameters `"-f GQ -cv -Ov"`. Further filtering was performed using vcftools (v.0.1.14)[22] (version 0.1.14) with settings `"--minQ 500 --minGQ 50 --minDP --max-alleles 2"`. As multiple samples used as part of this project correspond to single individuals, we calculated relatedness (vcftools --relatedness) and merged all alignment files corresponding to single individuals to increase genotyping sensitivity. The merged data corresponding to 143 individuals (comprising 243 datasets) was genotyped as above. Finally, variants where filtered that significantly deviated from Hardy-Weinberg equilibrium (HWE exact test; $p<0.01$). After filtering, 3,758,562 million SNVs heterozygous in one or more individuals remained.

Detecting allelic imbalance

For each sample, we filtered reads for which variants introduce mapping artifacts using the software package WASP[23]. Duplicate reads were discarded randomly. We determined allele-

specific read counts for all heterozygous position further filtering for number of alignment mismatches (>1 reference; >2 alternate) and genotype quality (>20). In addition, reads for which the variant position within 3 bp of the 5' end was discarded to avoid any variants that may affect DNase I cleavage rates.

We used beta-binomial distribution to test for imbalance as it allows for additional parameter to model dispersion. To tune the parameters of distribution we selected a set high confidence SNVs (n=407,511) with strong statistical power (heterozygous in 2 or more samples, at least 20 reads covering either allele in each sample and at least 100 total reads summed over all samples) and for each SNV, we computed the mean and standard deviation of the allelic ratio across all samples. We set the parameters α and β of the beta-binomial using the following equations:

$$\alpha = \mu \times \left(\frac{1}{\sigma^2} - 1\right)$$

$$\beta = (1 - \mu) \times \left(\frac{1}{\sigma^2} - 1\right) \quad \textit{Eq. 10}$$

where μ and σ correspond to the average of the means and standard deviations over all high confidence SNVs.

For each SNV containing ≥35 total summed reads over all heterozygous samples (from either allele) computed statistical significance of allelic imbalance using the Beta-binomial distribution (parameterized as above). Overall, 1,656,597 variants were tested for allelic imbalance. Multiple testing correction of the Beta-binomial *p*-values was performed using the Benjamini-Hochberg method. Due to the extremely conservative nature of our test, imbalanced variants were established at a false discovery rate of 20%.

Enrichment of imbalanced variants within TF recognition sequences

To assess the relative enrichment if imbalanced SNVs with respect to TF recognition sequences, we used an approach identical to ref. 15. Briefly, all tested variants were aligned relative to all motif models distinguishing whether each motif instance overlapped a consensus footprint. The proportion of SNVs imbalanced was computed using variants with an FDR<20% and reference allelic ratio ≥70%. Motifs were filtered to have ≥40 SNVs per position and at least 3 position with ≥7 imbalanced SNVs. Statistical significance of the enrichment was determined by permutation of the imbalance labels with respect to all SNVs within the motif and 20bp flanking regions. Multiple testing correction was performed using the Benjamini-Hochberg method and motifs were considered significant at a false discovery rate of 5%.

Energetic effects of variation on TF recognition sequences

To measure the effects of sequence variation within putative TF bindings sites with respect to allelic imbalance, we first created a custom genome containing all possible alleles by encoding variant positions using ambiguous IUPAC DNA codes (*e.g.*, `A/T=W`). We then identified motif matches within the custom genome using the variant aware mode of MOODS (v1.9.3)[14] (see

Transcription factor binding site predictions) that scans all possible alleles at ambiguous positions. For each variant that overlapped a motif instance, we computed a motif match score separately for each allele computed the log difference between the two scores.

Human genetic variation

Estimates of human population genetic variation was obtained from NHLBI TOPMED project (freeze 5), downloaded from the Bravo webserver (http://bravo.sph.umich.edu). Only variants passing all filters ("PASS") were considered. Indels were removed from all analysis. Nucleotide diversity measurements (π) were calculated from the TOPMED allele frequencies for each position genome-wide using the following equation:

$$\pi_i = 1 - \sum_{j=1}^{n} p_{i,j}^2 \qquad \textit{Eq. 11}$$

where $p_{i,j}$ is allele population frequency of a minor allele j at genomic position i. We estimated 95% confidence intervals of the mean of π by performing 1,000 bootstrap samples. Four-fold degenerate codings sites were defined using NCBI called open reading frames and filtered against RepeatMasked regions.

Deriving expected density of rare variants

To generate the expected density of rare variation within and around footprints, we utilized a published 7-mer mutation rate sequence model generated rare singleton variants in a large human corhort (>3,500)[24]. The 7-mer model encodes context information surrounding each possible mutation (*e.g.*, `NNN[C>G]NNN`, `NNN[C>T]NNN`, etc.). For each base pair in a ±100bp window centered on each consensus footprint, we computed the number of expected mutations by summing over the mutation rates of all three possible mutational outcomes (per 7-mer). The aggregate density of rare SNVs (Fig 3d; bottom) was computed by summing the expected mutation at each nucleotide (relative to footprint center), at consensus footprints and dividing by the median across the window.

GWAS variation

Disease- and trait-associated human genetic variation was obtained from the NHGRI-EBI GWAS Catalog[25] (v1.0 all associations) (https://www.ebi.ac.uk/gwas/docs/file-downloads). The enrichment of GWAS data within DHS or footprints was performed by an empirical sampling approach. Random SNVs from the 1,000 Genomes Project[20] (1KGP) (central European population) were sampled and overlapped with DHS or DNase I footprints. Sampled 1KGP SNVs were matched with observed GWAS SNVs for allele frequency and linkage disequilibrium structure (total number of SNVS with r^2=1 with GWAS allele). Both the GWAS and sampled SNPs were expanded to all SNVs in perfect LD before assessing overlaps with either DHS peaks or footprints. The sampling procedure was repeated 1,000 times to estimate the parameters of a normal distribution (μ, σ). These parameters were used to calculate the upper-tail *p*-value of the observed overlap of GWAS SNPs. To consider the effect of footprint threshold on GWAS enrichment, we established consensus footprints are various posterior probability

thresholds. The DHS peaks minus footprint regions were defined by the total genomic space covered by a DHS removing the positions corresponding to consensus footprints defined at a posterior probability of 95%.

Stratified LD-score regression analysis

SNP-based trait heritability was computed using LD-score regression (S-LDSC v1.0.0)[26,27] using a reference set of HapMap3 SNPs. In addition to the baseline set of 97 annotations provided as part of the LDSC software package (baseline-LD model v2.2; https://data.broadinstitute.org/alkesgroup/LDSCORE/), we created additional SNP annotations with respect to DHS and footprints. To generate these additional annotations, we merged all DHS or footprints with bedops[17] and lifted over their genomic coordinates to human genome build hg19 using CrossMap[28] with default parameters. GWAS summary statistics for two UK BioBank traits were download from (http://www.nealelab.is/uk-biobank; Benjamin Neale lab) corresponding to RBC count (30010_irnt.gwas.imputed_v3.both_sexes) and lymphocyte count (30120_irnt.gwas.imputed_v3.both_sexes). LDSC was run for each DHS or footprint annotation independently (each run including the 97 baseline annotations).

Supplementary References

1. Thurman, R. E. *et al.* The accessible chromatin landscape of the human genome. *Nature* **489**, 75–82 (2012).

2. Consortium, R. E. *et al.* Integrative analysis of 111 reference human epigenomes. *Nature* **518**, 317–330 (2015).

3. John, S. *et al.* Genome-scale mapping of DNase I hypersensitivity. *Current protocols in molecular biology* **Chapter 27**, Unit 21.27-21.27.20 (2013).

4. Li, H. & Durbin, R. Fast and accurate short read alignment with Burrows-Wheeler transform. *Bioinformatics* **25**, 1754–1760 (2009).

5. Vierstra, J. & Stamatoyannopoulos, J. A. Genomic footprinting. *Nat. Methods* **13**, 213–221 (2016).

6. Lazarovici, A. *et al.* Probing DNA shape and methylation state on a genomic scale with DNase I. *Proc. Natl. Acad. Sci. U. S. A.* **110**, 6376–6381 (2013).

7. Derrien, T. *et al.* Fast computation and applications of genome mappability. *PloS ONE* **7**, e30377 (2012).

8. Kent, W. J. *et al.* The Human Genome Browser at UCSC. *Genome Res.* **12**, 996–1006 (2002).

9. Meuleman, W. *et al.* Index and biological spectrum of accessible DNA elements in the human genome. *Nature* (2020).

10. Cooper, G. M. *et al.* Distribution and intensity of constraint in mammalian genomic sequence. *Genome Res.* **15**, 901–913 (2005).

11. Jolma, A. *et al.* DNA-binding specificities of human transcription factors. *Cell* **152**, 327–339 (2013).

12. Khan, A. *et al.* JASPAR 2018: update of the open-access database of transcription factor binding profiles and its web framework. *Nucleic Acids Res.* **46**, D1284 (2018).

13. Kulakovskiy, I. V. *et al.* HOCOMOCO: towards a complete collection of transcription factor binding models for human and mouse via large-scale ChIP-Seq analysis. *Nucleic Acids Res.* **46**, D252–D259 (2018).

14. Korhonen, J. H., Palin, K., Taipale, J. & Ukkonen, E. Fast motif matching revisited: high-order PWMs, SNPs and indels. *Bioinformatics* **33**, 514–521 (2017).

15. Maurano, M. T. *et al.* Large-scale identification of sequence variants influencing human transcription factor occupancy in vivo. *Nat. Genet.* **47**, 1393–1401 (2015).

16. Gupta, S., Stamatoyannopoulos, J. A., Bailey, T. L. & Noble, W. S. Quantifying similarity between motifs. *Genome Biol.* **8**, R24 (2007).

17. Neph, S. *et al.* BEDOPS: high-performance genomic feature operations. *Bioinformatics* **28**, 1919–1920 (2012).

18. Yin, M. *et al.* Molecular mechanism of directional CTCF recognition of a diverse range of genomic sites. *Cell Res.* **27**, 1365–1377 (2017).

19. Xu, H. E. *et al.* Crystal structure of the human Pax6 paired domain-DNA complex reveals specific roles for the linker region and carboxy-terminal subdomain in DNA binding. *Genes Dev.* **13**, 1263–1275 (1999).

20. Li, S., Olson, W. K. & Lu, X.-J. Web 3DNA 2.0 for the analysis, visualization, and modeling of 3D nucleic acid structures. *Nucleic Acids Res.* **47**, W26–W34 (2019).

21. The PyMOL Molecular Graphics System, Version~1.8. (2015).

22. Danecek, P. *et al.* The variant call format and VCFtools. *Bioinformatics* **27**, 2156–2158 (2011).

23. Geijn, B. van de, McVicker, G., Gilad, Y. & Pritchard, J. K. WASP: allele-specific software for robust molecular quantitative trait locus discovery. *Nat. Methods* **12**, 1061–3 (2015).

24. Carlson, J. *et al.* Extremely rare variants reveal patterns of germline mutation rate heterogeneity in humans. *Nat. Commun.* **9**, 3753 (2018).

25. Buniello, A. *et al.* The NHGRI-EBI GWAS Catalog of published genome-wide association studies, targeted arrays and summary statistics 2019. *Nucleic Acids Res.* **47**, D1005–D1012 (2019).

26. Bulik-Sullivan, B. K. *et al.* LD Score regression distinguishes confounding from polygenicity in genome-wide association studies. *Nat. Genet.* **47**, 291–295 (2015).

27. Finucane, H. K. *et al.* Partitioning heritability by functional annotation using genome-wide association summary statistics. *Nat. Genet.* **47**, 1228–1235 (2015).

28. Zhao, H. *et al.* CrossMap: a versatile tool for coordinate conversion between genome assemblies. *Bioinformatics* **30**, 1006–1007 (2014).
