# Supplementary material for: Global reference mapping of human transcription factor footprints
Source: Nature. 2020 Jul 29;583(7818):729–36. doi: 10.1038/s41586-020-2528-x (PMC7410829; doi:10.1038/s41586-020-2528-x)
Supplement: Supplementary file 2 — Reporting Summary [file 41586_2020_2528_MOESM2_ESM.pdf]

nature research

Corresponding author(s): Jeff Vierstra and John A. Stamatoyannopoulos

Last updated by author(s): May 28, 2020

Reporting Summary

Nature Research wishes to improve the reproducibility of the work that we publish. This form provides structure for consistency and transparency in reporting. For further information on Nature Research policies, see our Editorial Policies and the Editorial Policy Checklist.

Statistics

For all statistical analyses, confirm that the following items are present in the figure legend, table legend, main text, or Methods section.

n/a	Confirmed	
☐	☒	The exact sample size (*n*) for each experimental group/condition, given as a discrete number and unit of measurement
☐	☒	A statement on whether measurements were taken from distinct samples or whether the same sample was measured repeatedly
☐	☒	The statistical test(s) used AND whether they are one- or two-sided<br>*Only common tests should be described solely by name; describe more complex techniques in the Methods section.*
☒	☐	A description of all covariates tested
☐	☒	A description of any assumptions or corrections, such as tests of normality and adjustment for multiple comparisons
☐	☒	A full description of the statistical parameters including central tendency (e.g. means) or other basic estimates (e.g. regression coefficient) AND variation (e.g. standard deviation) or associated estimates of uncertainty (e.g. confidence intervals)
☐	☒	For null hypothesis testing, the test statistic (e.g. *F*, *t*, *r*) with confidence intervals, effect sizes, degrees of freedom and *P* value noted<br>*Give P values as exact values whenever suitable.*
☒	☐	For Bayesian analysis, information on the choice of priors and Markov chain Monte Carlo settings
☐	☒	For hierarchical and complex designs, identification of the appropriate level for tests and full reporting of outcomes
☒	☐	Estimates of effect sizes (e.g. Cohen's *d*, Pearson's *r*), indicating how they were calculated

Our web collection on statistics for biologists contains articles on many of the points above.

Software and code

Policy information about availability of computer code

Data collection	Sequencing data was collected using an Illumina HiSeq 4000 and processed using HCS software v3.3.76 (Illumina, Inc.).
Data analysis	Data was analyzed using following software tools: bwa (v0.7.12), BEDOPS (v2.4.39), python (v2.7 and 3.6), GNU awk (v4.0.2), pyMOL (v2.3.2), WASP (v0.3.4), bcftools (v1.9), vcftools (v0.1.14), MOODS (v1.9.3), S-LDSC (v1.0.0). Custom software developed for this manuscript is freely available under an open-source MIT license at http://www.github.com/jvierstra/footprint-tools.

For manuscripts utilizing custom algorithms or software that are central to the research but not yet described in published literature, software must be made available to editors and reviewers. We strongly encourage code deposition in a community repository (e.g. GitHub). See the Nature Research guidelines for submitting code & software for further information.

Data

Policy information about availability of data

All manuscripts must include a data availability statement. This statement should provide the following information, where applicable:

- Accession codes, unique identifiers, or web links for publicly available datasets
- A list of figures that have associated raw data
- A description of any restrictions on data availability

All raw sequencing data and primary processing of the DNase I data is available through the ENCODE data portal (http://www.encodeproject.org/) with accessions listed in Extended Data Table 1. Footprints and associated analysis are available at http://vierstra.org/resources/dgf or https://doi.org/10.5281/zenodo.3603548 (ZENODO). A track hub to visualize data in the UCSC Genome Browser is hosted at https://resources.altius.org/~jvierstra/projects/footprinting.2020/hub.txt. Protein structures for CTCF (Fig. 2a) and PAX6 (Extended Data Fig. 6a) were downloaded from Protein Data Bank (https://www.rcsb.org/) (PDB ID: 5YEF, 5YEL and 6PAX).

Field-specific reporting

Please select the one below that is the best fit for your research. If you are not sure, read the appropriate sections before making your selection.

☒ Life sciences ☐ Behavioural & social sciences ☐ Ecological, evolutionary & environmental sciences

For a reference copy of the document with all sections, see nature.com/documents/nr-reporting-summary-flat.pdf

Life sciences study design

All studies must disclose on these points even when the disclosure is negative.

Sample size	No sample sizes calculations were performed -- all data was used where applicable
Data exclusions	No data was excluded from analysis
Replication	Not applicable -- no group-wise experimental testing was performed
Randomization	Not applicable -- no group-wise experimental testing was performed
Blinding	Not applicable -- no group-wise experimental testing was performed

Reporting for specific materials, systems and methods

We require information from authors about some types of materials, experimental systems and methods used in many studies. Here, indicate whether each material, system or method listed is relevant to your study. If you are not sure if a list item applies to your research, read the appropriate section before selecting a response.

Materials & experimental systems

n/a	Involved in the study	
☒	☐	Antibodies
☐	☒	Eukaryotic cell lines
☒	☐	Palaeontology and archaeology
☒	☐	Animals and other organisms
☒	☐	Human research participants
☒	☐	Clinical data
☒	☐	Dual use research of concern

Methods

n/a	Involved in the study	
☒	☐	ChIP-seq
☒	☐	Flow cytometry
☒	☐	MRI-based neuroimaging

Eukaryotic cell lines

Policy information about cell lines

Cell line source(s)	Cell lines were procured from appropriate commercial sources. h.ESC lines used were from NIH approved list and provided by laboratories with expertise in growing, characterizing and differentiating these cell types. (see ENCODE website for details and protocols).
Authentication	Cells lines were authenticated in accordance with ENCODE policies.
Mycoplasma contamination	Mycoplasma testing was not performed.
Commonly misidentified lines (See ICLAC register)	None
